# Guidance for protocol content and reporting of dog-assisted interventions in randomised controlled trials: explanation and elaboration of the SPIRIT 2025 and CONSORT 2025 extensions

**DOI:** 10.1186/s12874-025-02663-6

**Published:** 2026-05-14

**Authors:** Emily Shoesmith, Elena Ratschen, Evgenia Riga, Daniel S. Mills, Selina Gibsone, Dean McMillan, Qi Wu, Chris Clarke, Kirsty Sprange, Kerstin Meints, Nancy R. Gee, Leanne O. Nieforth, Nicolas Dollion, Aubrey Fine, Sophie S. Hall

**Affiliations:** 1 Department of Health Sciences, University of York, York, UK; 2 Department of Life Sciences, University of Lincoln, Lincoln, UK; 3 Dogs for Good, The Frances Hay Centre, Banbury, UK; 4 Tees, Esk and Wear Valleys, Foss Park Hospital, NHS Foundation Trust, York, UK; 5 Nottingham Clinical Trials Unit, School of Medicine, University of Nottingham, Nottingham, UK; 6 School of Psychology, Sports Science and Wellbeing, University of Lincoln, Lincoln, UK; 7 Center for Human-Animal Interaction, School of Medicine, Virginia Commonwealth University, Richmond, VA USA; 8 Center for the Human-Animal Bond, College of Veterinary Medicine, Purdue University, Lafayette, USA; 9 Université de Reims Champagne-Ardenne, Laboratoire C2S, UR6291, Reims, France; 10 Department of Education, California State Polytechnic University, Pomona, CA USA

**Keywords:** SPIRIT, CONSORT, Dog-Assisted interventions, Reporting guidelines

## Abstract

**Background:**

Dog-assisted interventions (DAIs) to improve human health and wellbeing are increasingly implemented in health, care and educational settings. However, the quality and consistency of reporting of trial design and findings remains poor. Current trial reporting guidelines do not adequately address the unique methodological, ethical, and welfare considerations involved in DAI randomised controlled trials (RCTs). To improve the design and transparent reporting of these trials, we developed SPIRIT (Standard Protocol Items: Recommendations for Interventional Trials) 2025 and CONSORT (Consolidated Standards of Reporting Trials) 2025 extensions for DAI trials. The current paper presents the explanation and elaboration (E&E) for these extensions.

**Methods:**

Using the Enhancing the Quality and Transparency of Health Research (EQUATOR) methodological framework, a four-phase consensus approach was conducted: (1) a systematic review; (2) three-round Delphi survey; (3) expert consensus workshops, and (4) checklist finalisation. This E&E paper complements the DAI extensions checklist (reported elsewhere) by providing item-by-item rationales, guidance, and illustrative examples from DAI trials to demonstrate good reporting.

**Results:**

The four-phase consensus approach resulted in 69 extensions for DAI RCT reporting (35 applied to SPIRIT; 34 applied to CONSORT). This paper explains the purpose of each extension item and highlights best practice for reporting, based on examples from published DAI protocols and trial reports.

**Conclusion:**

These DAI extensions provide guidance for the design and reporting of high-quality and transparent DAI RCTs. This E&E paper supports the application of these extensions by offering detailed explanations and examples. Adoption by researchers, journal editors, funders, and ethics committees may enhance the credibility and impact of DAI research.

## Introduction

The SPIRIT (Standard Protocol Items: Recommendations for Interventional Trials) and CONSORT (Consolidated Standards of Reporting Trials) statements were developed to facilitate the reporting of trial protocols and randomised controlled trials (RCTs), respectively [1–4]. Both statements were most recently updated in 2025 to reflect methodological advancements and incorporate feedback from end users, thereby ensuring its continued relevance and use in guiding the reporting of protocols and RCTs [5, 6]. The SPIRIT 2025 and CONSORT 2025 statements have improved the overall reporting quality of RCT protocols [7] and RCT reports [8–11]. These statements have been endorsed by thousands of journals on a global scale [12], are accessible in different languages [13], and are frequently listed as a requirement for manuscript submission [12, 14].

The SPIRIT 2025 and CONSORT 2025 statements primarily focus on individually randomised two-group parallel trials [4]. To address the varying amount of additional information required for various trial types, extensions of the SPIRIT and CONSORT statements have been created for different types of trial designs, for example, cluster randomised trials [15], non-inferiority and equivalence trials [16], crossover trials [17], and stepped wedge trials [18]. Additionally, intervention extensions of the SPIRIT and CONSORT statements have been developed, for example, for non-pharmacological [19] and social and psychological interventions [20]. These extensions were developed to overcome commonly cited limitations of non-pharmacological interventions, as several reviews have reported they are often inadequately or inaccurately reported, and lack the transparency required for replication and quality assessment [21–25]. These limitations can contribute to inadequate dissemination of effective interventions [26, 27], overestimation of intervention efficacy [28] and research waste [26].

Although the SPIRIT and CONSORT statements and their relevant extensions promote the transparent and detailed reporting of social and psychological interventions, requirements for the transparent reporting of dog-assisted interventions (DAIs) arguably go beyond the scope of guidelines that are currently available. While many of the methodological and ethical considerations described in this paper may be relevant to other animal-assisted interventions (AAIs), we chose to focus exclusively on DAIs as they are the most commonly provided and researched type of AAI [29]. This specificity allows for tailored guidance that reflects current practice and supports the standardisation of DAI research. DAIs often use concepts that are distinct from those used by researchers targeted by the SPIRIT and CONSORT statements and/or relevant extensions. DAI research involves a unique set of design features and considerations that are not addressed in the standard statements. Examples include DAI-specific details relating to intervention (e.g., goal orientation for outcomes including suggested mechanisms of action; choice/training/selection of dog and human provider teams); recruitment (e.g., identifying suitable participant and dog characteristics); and assessing/managing harms (e.g., safety of participant-dog interactions including monitoring of dog wellbeing/welfare). The need for robust research programmes to improve the evidence base in the area and reliably inform DAI practice has been highlighted [30, 31].

To facilitate improved reporting, we developed SPIRIT and CONSORT extensions for reporting DAIs in RCTs [under review]. The current report serves as an accompanying Explanation & Elaboration (E&E) paper. This E&E paper presents each SPIRIT and CONSORT extension for DAI RCTs, along with a detailed rationale and examples of good reporting. For the purpose of guidance, DAIs refer to therapeutic, educational, or activity-based interventions involving dogs and do not include service dog placements. Examples of DAIs include, but are not limited to, structured therapy sessions in healthcare settings educational programmes within schools, assisted activities in residential care facilities, and visitation initiatives in community centres or prisons. We also acknowledge that the term ‘canine-assisted intervention’ is used in the literature and is sometimes used interchangeably with DAIs. However, for clarity and consistency, we use DAIs throughout this manuscript.

## Explanation and Elaboration of the SPIRIT 2025 and CONSORT 2025 extensions

This E&E paper is intended to be used in conjunction with the SPIRIT 2025 and CONSORT 2025 statements [5, 6], as well as the extensions for DAI RCTs [under review]. For clarity, the primary focus is on DAIs, although most of the recommendations could extend to AAIs involving non-dog species, as many of the methodological, ethical, and welfare considerations (e.g., handler training, animal wellbeing, participant safety and intervention design) are applicable across species regardless of the specific animal involved. The SPIRIT and CONSORT checklists for DAIs were developed concurrently, and the methods have been described in detail elsewhere [under review]. However, in brief, this included a four-phase consensus approach, including: 1) a systematic review to identify key reporting considerations in DAI RCTs to develop a ‘long list’ of SPIRIT and CONSORT extension items [31]; 2) a three-round Delphi survey to refine the ‘long list’ of extension items; 3) consensus workshops with academic experts, all of whom held doctoral degrees and possessed over ten years of experience in their respective fields. Six participants had internationally recognised track records in human-animal interaction research, specifically in DAI research (*n* = 6), and one participant was an experienced methodologist with expertise in clinical trial design and reporting, who also held a research interest in this area; and 4) checklist finalisation involving iterative revisions and piloting of extension items and supporting E&E statements. The standard SPIRIT and CONSORT checklists are presented in Tables 1 and 2, respectively, with the suggested extensions for DAIs.

**Table 1:** Checklist for reporting of DAIs: extension of the SPIRIT 2025 statement

**Section/topic**	No	SPIRIT 2013 checklist item	Extension for DAI RCTs
**Administrative information**			
Title and structured summary	1a	Title stating the trial design, population, and interventions, with identification as a protocol	The species of animal involved (i.e., dog)
			Type of DAI (e.g., therapy, activity, education)
	1b	Structured summary of trial design and methods, including items from the World Health Organization Trial Registration Data Set	N/A
Protocol version	2	Version date and identifier	
Roles and responsibilities	3a	Names, affiliations, and roles of protocol contributors	N/A
	3b	Name and contact information for the trial sponsor	N/A
	3c	Role of trial sponsor and funders in design, conduct, analysis, and reporting of trial; including any authority over these activities	N/A
	3d	Composition, roles, and responsibilities of the coordinating site, steering committee, endpoint adjudication committee, data management team, and other individuals or groups overseeing the trial, if applicable	Roles and responsibilities of individual(s) responsible for dog welfare
			State which DAI guidelines/code of practice are used if appropriate
			Name, accreditation status, non-/for-profit status of any animal-assisted intervention organisations involved in intervention (where applicable)
**Open Science**			
Trial registration	4	Name of trial registry, identifying number (with URL), and date of registration. If not yet registered, name of intended registry	N/A
Protocol and statistical analysis plan	5	Where the trial protocol and statistical analysis plan can be accessed	N/A
Data sharing	6	Where and how the individual de-identified participant data (including data dictionary), statistical code, and any other materials will be accessible	N/A
Funding and conflicts of interest	7a	Sources of funding and other support (for example, supply of drugs)	N/A
	7b	Financial and other conflicts of interest for principal investigators and steering committee member	N/A
Dissemination policy	8	Plans to communicate trial results to participants, healthcare professionals, the public, and other relevant groups (for example, reporting in trial registry, plain language summary, publication)	N/A
**Introduction**			
Background and rationale	9a	Scientific background and rationale, including summary of relevant studies (published and unpublished) examining benefits and harms for each intervention	Scientific background and rationale for including a DAI
			Description of proposed mechanism(s), model(s) or theories describing the potential impact of the DAI, if applicable
	9b	Explanation for choice of comparator	N/A
Objectives	10	Specific objectives related to benefits and harms	Specific objectives or hypotheses related to the DAI
**Methods: Patient and public involvement, trial design**			
Patient and public involvement	11	Details of, or plans for, patient or public involvement in the design, conduct, and reporting of the trial	N/A
Trial design	12	Description of trial design including type of trial (for example, parallel group, crossover), allocation ratio, and framework (for example, superiority, equivalence, non-inferiority, exploratory)	N/A
**Methods: Participants, interventions and outcomes**			
Trial setting	13	Settings (for example, community, hospital) and locations (for example, countries, sites) where the trial will be conducted	Description of how considerations related to dog welfare are incorporated in the study context and settings
			Description of the characteristics of study settings and the DAI that may affect dogs (e.g., room size, noises, smells, lighting)
			Description of how the suitability of the dog for the DAI in the given study context and environment is ensured
Eligibility criteria	14a	Eligibility criteria for participants	Eligibility criteria relevant to engaging/interacting with dog, if any (e.g., dog phobia, allergies, history of dog abuse)
	14b	If applicable, eligibility criteria for sites and for individuals who will deliver the interventions (for example, surgeons, physiotherapists)	Description of any criteria for sites to ensure dog welfare requirements can be met
			Selection criteria for the dog(s) involved in the intervention and justification for these
			Description of relevant training and education undertaken by all parties that is pertinent to their role in the intervention (e.g., participant vs. dog handler). This may include training on dog welfare, safety and intervention-specific content
Intervention and comparator	15a	Intervention and comparator with sufficient details to allow replication including how, when, and by whom they will be administered. If relevant, where additional materials describing the intervention and comparator (for example, intervention manual) can be accessed	Description of DAI goal and content
			Description of the proposed duration and frequency of DAI including, where possible, justification
			Description of tasks and roles of each individual in the DAI team (i.e., dog, dog handler, other trained professional) including details of participant-dog interactions
			Where possible, consideration of suitability/tailoring of DAI under EDI aspects (e.g., severity of illness)
			Rationale for and description of the comparator group
	15b	Criteria for discontinuing or modifying allocated intervention/comparator for a trial participant (for example, drug dose change in response to harms, participant request, or improving/worsening disease)	Criteria and/or processes for discontinuing or modifying the intervention based on dog and/or handler responses
	15c	Strategies to improve adherence to intervention/comparator protocols, if applicable, and any procedures for monitoring adherence (for example, drug tablet return, sessions attended)	N/A
	15d	Concomitant care that is permitted or prohibited during the trial	Description of whether and how potential concomitant human–dog interactions (e.g., with pet dog) are controlled for
Outcomes	16	Primary and secondary outcomes, including the specific measurement variable (for example, systolic blood pressure), analysis metric (for example, change from baseline, final value, time to event), method of aggregation (for example, median, proportion), and time point for each outcome	Outcome measures relevant to the proposed pathway of action of the DAI
			Where applicable, justify the use of unblinded outcome measures in relation to the DAI
Harms	17	How harms are defined and assessed (for example, systematically, non-systematically)	Observations/measures relevant to the impact of the intervention on the dog(s)
Participant timeline	18	Time schedule of enrolment, interventions (including any run-ins and washouts), assessments, and visits for participants. A schematic diagram is highly recommended	Time schedule of any required participant-dog training/familiarity sessions prior to commencement of the intervention
Sample size	19	How sample size was determined, including all assumptions supporting the sample size calculation	Description of capacity of dog-handler teams required to safely and effectively deliver the intervention
Recruitment	20	Strategies for achieving adequate participant enrolment to reach target sample size	N/A
**Methods: Assignment of interventions**			
Sequence generation	21a	Who will generate the random allocation sequence, and the method used	If possible, describe the process of matching dog-handler teams (dog & handler) to participants, including the individual/team responsible for this process, where relevant
	21b	Type of randomization (simple or restricted) and details of any factors for stratification. To reduce predictability of a random sequence, other details of any planned restriction (for example, blocking) should be provided in a separate document that is unavailable to those who enrol participants or assign interventions	Description of whether concomitant interactions with dogs (e.g., interactions with pet/other dogs) is included as a stratification variable
Allocation concealment mechanism	22	Mechanism used to implement the random allocation sequence (for example, central computer/telephone; sequentially numbered, opaque, sealed containers), describing any steps to conceal the sequence until interventions are assigned	N/A
Implementation	23	Whether the personnel who will enrol and those who will assign participants to the interventions will have access to the random allocation sequence	N/A
Blinding	24a	Who will be blinded after assignment to interventions (for example, participants, care providers, outcome assessors, data analysts)	Description and justification of whether trained professional(s) and dog handler(s) are blinded to the outcomes and how
	24b	If blinded, how blinding will be achieved and description of the similarity of interventions	N/A
	24c	If blinded, circumstances under which unblinding is permissible, and procedure for revealing a participant’s allocated intervention during the trial	N/A
**Methods: Data collection, management, and analysis**			
Data collection methods	25a	Plans for assessment and collection of trial data, including any related processes to promote data quality (for example, duplicate measurements, training of assessors) and a description of trial instruments (for example, questionnaires, laboratory tests) along with their reliability and validity, if known. Reference to where data collection forms can be accessed, if not in the protocol	N/A
	25b	Plans to promote participant retention and complete follow-up, including list of any outcome data to be collected for participants who discontinue or deviate from intervention protocols	N/A
Data management	26	Plans for data entry, coding, security, and storage, including any related processes to promote data quality (for example, double data entry; range checks for data values). Reference to where details of data management procedures can be accessed, if not in the protocol	N/A
Statistical methods	27a	Statistical methods used to compare groups for primary and secondary outcomes, including harms	N/A
	27b	Definition of who will be included in each analysis (for example, all randomized participants), and in which group	N/A
	27c	How missing data will be handled in the analysis	N/A
	27d	Methods for any additional analyses (for example, subgroup and sensitivity analyses)	Explain whether a subgroup analysis considers dog characteristics (e.g., size)
**Methods: Monitoring**			
Data monitoring committee	28a	Composition of data monitoring committee (DMC); summary of its role and reporting structure; statement of whether it is independent from the sponsor and funder; conflicts of interest and reference to where further details about its charter can be found, if not in the protocol. Alternatively, an explanation of why a DMC is not needed	Summary of the role of the DMC or other relevant committees in relation to monitoring dog welfare
	28b	Explanation of any interim analyses and stopping guidelines, including who will have access to these interim results and make the final decision to terminate the trial	Justification, and description, of whether interim analyses and stopping guidelines consider dog welfare
Trial monitoring	29	Frequency and procedures for monitoring trial conduct. If there is no monitoring, give explanation	Plans for collecting, assessing, reporting and managing harms or unintended effects for the dog(s)
**Ethics**			
Research ethics approval	30	Plans for seeking research ethics committee/institutional review board approval	N/A
Protocol amendments	31	Plans for communicating important protocol modifications to relevant parties	N/A
Consent or assent	32a	Who will obtain informed consent or assent from potential trial participants or authorized proxies, and how	N/A
	32b	Additional consent provisions for collection and use of participant data and biological specimens in ancillary studies, if applicable	N/A
Confidentiality	33	How personal information about potential and enrolled participants will be collected, shared, and maintained in order to protect confidentiality before, during, and after the trial	N/A
Ancillary and post-trial care	34	Provisions, if any, for ancillary and post-trial care, and for compensation to those who suffer harm from trial participation	Description for post-session and/or post-intervention care in relation to dog wellbeing where applicable
			Provisions for supporting the end of interactions between the dog and participant (post-session and/or post-intervention)

**Table 2:** Checklist for reporting of DAIs: extension of the CONSORT 2025 statement

**Section/topic**	No	CONSORT 2025 checklist item	Extension for DAI RCTs
**Title and abstract**			
Title and structured abstract	1a	Identification as a randomised trial	The species of animal involved (i.e., dog)
			Type of DAI (e.g., therapy, activity, education)
	1b	Structured summary of trial design, methods, results, and conclusions	N/A
**Open science**			
Trial registration	2	Name of trial registry, identifying number (with URL) and date of registration	N/A
Protocol and statistical analysis plan	3	Where the trial protocol and statistical analysis plan can be accessed	N/A
Data sharing	4	Where and how the individual de-identified participant data (including data dictionary), statistical code and any other materials can be accessed	N/A
Funding and conflicts of interest	5a	Sources of funding and other support (e.g., supply of drugs), and role of funders in the design, conduct, analysis, and reporting of the trial	N/A
	5b	Financial and other conflicts of interest of the manuscript authors	Name, accreditation status, non-/for-profit status of any animal-assisted intervention organisations involved in intervention (where applicable)
**Introduction**			
Background and rationale	6	Scientific background and rationale	Scientific background and rationale for including a DAI
			Description of proposed mechanism(s), model(s), or theories describing the potential impact of the DAI, if applicable
Objectives	7	Specific objectives related to benefits and harms	Specific objectives or hypotheses related to the DAI
**Methods**			
Patient and public involvement	8	Details of patient or public involvement in the design, conduct and reporting of the trial	N/A
Trial design	9	Description of trial design including type of trial (e.g., parallel group, crossover), allocation ratio, and framework (e.g., superiority, equivalence, non-inferiority, exploratory)	N/A
Changes to trial protocol	10	Important changes to the trial after it commenced including any outcomes or analyses that were not prespecified, with reason	N/A
Trial setting	11	Settings (e.g., community, hospital) and locations (e.g., countries, sites) where the trial was conducted	Description of how considerations related to dog welfare are incorporated in the study context and settings
			Description of the characteristics of study settings and the DAI that may affect dogs (e.g., room size, noises, smells, lighting)
			Description of how the suitability of the dog for the DAI in the given study context and environment is ensured
Eligibility criteria	12a	Eligibility criteria for participants	Eligibility criteria relevant to engaging/interacting with dog, if any (e.g., dog phobia, allergies, history of dog abuse)
	12b	If applicable, eligibility criteria for sites and for individuals delivering the interventions (e.g., surgeons, physiotherapists)	Selection criteria for the dog(s) involved in the intervention and justification for these
			Description of any criteria for sites to ensure dog welfare requirements can be met
			Description of relevant training and education undertaken by all parties that is pertinent to their role in the intervention (e.g., participant vs. dog handler). This may include training on dog welfare, safety and intervention-specific content
Intervention and comparator	13	Intervention and comparator with sufficient details to allow replication. If relevant, where additional materials describing the intervention and comparator (e.g., intervention manual) can be accessed	Roles and responsibilities of individual(s) responsible for animal welfare
			State which DAI guidelines/code of practice are used if appropriate
			Description of DAI goal and content
			Description of the proposed duration and frequency of DAI including, where possible, justification
			Description of tasks and roles of each individual in the DAI team (i.e., dog, dog handler, other trained professional) including details of participant-dog interactions
			Where possible, consideration of suitability/tailoring of DAI under equality, diversity and inclusion aspects (e.g., severity of illness)
			Rationale for and description of the comparator group
Outcomes	14	Prespecified primary and secondary outcomes, including the specific measurement variable (e.g., systolic blood pressure), analysis metric (e.g., change from baseline, final value, time to event), method of aggregation (e.g., median, proportion), and time point for each outcome	Outcome measures relevant to the proposed pathway of action of the DAI
			Where applicable, justify the use of unblinded outcome measures in relation to the DAI
Harms	15	How harms were defined and assessed (e.g., systematically, non-systematically)	Observations/measures relevant to the impact of the intervention on the dog(s)
Sample size	16a	How sample size was determined	Description of capacity of dog-handler teams required to safely and effectively deliver the intervention
	16b	Explanation of any interim analyses and stopping guidelines	Justification, and description, of whether interim analyses and stopping guidelines consider dog welfare
Sequence generation	17a	Who generated the random allocation sequence, and the method used	N/A
	17b	Type of randomisation and details of any restriction (e.g., stratification, blocking, and block size)	Description of whether concomitant interactions with dogs (e.g., interactions with pet/other dogs) is included as a stratification variable
Allocation concealment mechanism	18	Mechanism used to implement the random allocation sequence (e.g., central computer/telephone; sequentially numbered, opaque, sealed containers), describing any steps to conceal the sequence until interventions were assigned	N/A
Implementation	19	Whether the personnel who enrolled and those who assigned participants to the interventions had access to the random allocation sequence	If possible, describe the process of matching dog-handler teams (dog & handler) to participants, including the individual/team responsible for this process, where relevant
Blinding	20a	Who was blinded after assignment to interventions (e.g., participants, care providers, outcome assessors, data analysts)	Description and justification of whether trained professional(s) and dog handler(s) are blinded to the outcomes and how
	20b	If blinded, how blinding was achieved and description of the similarity of interventions	N/A
Statistical methods	21a	Statistical methods used to compare groups for primary and secondary outcomes, including harms	N/A
	21b	Definition of who is included in each analysis (e.g., all randomised participants), and in which group	N/A
	21c	How missing data were handled in the analysis	N/A
	21d	Methods for any additional analyses (e.g., subgroup and sensitivity analyses), distinguishing prespecified from post hoc	N/A
**Results**			
Participant flow, including flow diagram	22a	For each group, the numbers of participants who were randomly assigned, received intended intervention, and were analysed for the primary outcome	Time schedule and completion rates of any required participant-dog training/familiarity sessions prior to commencement of the intervention
	22b	For each group, losses and exclusions after randomisation, together with reasons	Participant losses/exclusions for reasons relating to interacting with a dog
			Losses/exclusions of the dog(s)/dog handler(s) with reasons why
Recruitment	23a	Dates defining the periods of recruitment and follow-up for outcomes of benefits and harms	N/A
	23b	If relevant, why the trial ended or was stopped	N/A
Intervention and comparator delivery	24a	Intervention and comparator as they were actually administered (e.g., where appropriate, who delivered the intervention/comparator, whether participants adhered, whether they were delivered as intended (fidelity))	N/A
	24b	Concomitant care received during the trial for each group	N/A
Baseline data	25	A table showing baseline demographic and clinical characteristics for each group	Baseline data indicating concomitant interactions with dogs (e.g., interactions with pets/other dogs) if relevant
			Description of the number and characteristics of the dog(s) and their handler(s) involved
Numbers analysed, outcomes, and estimation	26	For each primary and secondary outcome, by group: • the number of participants included in the analysis • the number of participants with available data at the outcome time point • result for each group, and the estimated effect size and its precision (such as 95% confidence interval) • for binary outcomes, presentation of both absolute and relative effect size	N/A
Harms	27	All important harms or unintended effects in each group (for specific guidance, see CONSORT for harms)	Harms or unintended effects associated with the DAI, for the dog, and how these were assessed
Ancillary analyses	28	Any other analyses performed, including subgroup and sensitivity analyses, distinguishing prespecified from post hoc	Explain whether a subgroup analysis considers dog characteristics (e.g., size)
**Discussion**			
Interpretation	29	Interpretation consistent with results, balancing benefits and harms, and considering other relevant evidence	N/A
Limitations	30	Trial limitations, addressing sources of potential bias, imprecision, generalisability, and, if relevant, multiplicity of analyses	N/A

In the following section, we discuss each extension checklist item, explain the rationale, and provide an example of good reporting. CONSORT examples were mainly identified from RCTs included in the systematic review previously conducted by the team to identify CONSORT limitations in DAI RCT research [31]. In addition, we conducted database searches and registry searches (e.g., International Standard Randomised Controlled Trial Number; Clinicaltrials.gov), which identified relevant protocol papers in this area (*n* = 8). Examples are presented first to illustrate good reporting practice, followed by an explanation of the rationale behind each extension. The examples provided are not intended as prescriptive standards, but rather as practical illustrations to support interpretation and application of extension items. Examples are not exhaustive of all DAI types and may not be universally representative; their role is to support readers in applying guidance across diverse DAI types, including, for example, dog-assisted therapy, dog-assisted activities, and dog-assisted educational programmes.

Related items from the SPIRIT and CONSORT checklists (e.g., SPIRIT item 1a and CONSORT item 1a, both pertaining to the title) are grouped together, with the DAI-specific extension listed beneath the original item. We provide a single explanation that applies to both SPIRIT and CONSORT items, and examples of good reporting were selected based on the quality of reporting of the DAI-specific extension.

### SPIRIT 1a: Title stating the trial design, population, and interventions, with identification as a protocol

### CONSORT 1a: Identification as a randomised trial

- ➢ **Extension for DAI trials: The species of animal involved (i.e., dog)**

*SPIRIT example: “*Effects of dog-assisted therapy on the physical function and communication skills of adults with autism: A study protocol for a controlled study.” [32].

*CONSORT example: “*Dog‐assisted therapy vs relaxation for children and adolescents with Fetal Alcohol Spectrum Disorder: A randomized controlled study.” [33].

*Explanation:* Animal-assisted interventions can involve various animal species, e.g., dogs, horses, farm animals and small mammals [34–37]. Depending on the species involved, human-animal interaction type, expected benefits, and other aspects, such as participant engagement [38], can vary. Including the animal species involved in the title facilitates rapid identification of relevant articles when searching databases.

### SPIRIT 1a: Title stating the trial design, population, and interventions, with identification as a protocol.

### CONSORT 1a: Identification as a randomised trial

- ➢ **Extension for DAI trials: Type of dog-assisted intervention (DAI) (e.g., therapy, activity, education).**

*SPIRIT example: “*Effects of dog-assisted therapy on the physical function and communication skills of adults with autism: A study protocol for a controlled study.” [32].

*CONSORT example: “*Dog‐assisted therapy vs relaxation for children and adolescents with Fetal Alcohol Spectrum Disorder: A randomized controlled study.” [33].

*Explanation:* The type of dog-assisted intervention (DAI) chosen defines the purpose, structure and scope of the interaction between participants and the dog involved [39, 40]. DAIs typically encompass dog-assisted therapy (DAT), dog-assisted activities (DAA), and dog-assisted education (DAE) [41]. Clearly identifying the type of intervention promotes clarity and relevance for stakeholders (e.g., DAI providers, healthcare/educational professionals, academics, participants). There has been a recent suggestion for new terminologies (animal-assisted services; AASs [42]), but these have yet to be widely accepted.

### SPIRIT 3d: Composition, roles, and responsibilities of the coordinating site, steering committee, endpoint adjudication committee, data management team, and other individuals or groups overseeing the trial, if applicable.

### CONSORT 13: Intervention and comparator with sufficient details to allow replication. If relevant, where additional materials describing the intervention and comparator (e.g., intervention manual) can be accessed

The following two DAI extensions apply to these SPIRIT and CONSORT items.

- ➢ **Extension for DAI trials: Roles and responsibilities of individual(s) responsible for dog welfare.**

*SPIRIT example:* “The therapy dog will be owned and cared for by the treating therapist. The therapist will take responsibility for correct hygiene and grooming of the dog.” [43].

*CONSORT example:* “In the present study, the sessions were conducted by technicians in AAT. They were also responsible for covering all the dogs’ needs and for ensuring they were in good conditions of health and hygiene, thus guaranteeing correct treatment of residents and animals.” [44].

*Explanation:* Ensuring the welfare of dogs involved in DAIs is essential for ethical and effective practice. Clearly defining the roles and responsibilities of individuals overseeing dog welfare helps to safeguard the dogs’ wellbeing, ensures compliance with ethical guidelines, and facilitates the identification of the person responsible for dog welfare practices to external and internal parties.

- ➢ **Extension for DAI trials: State which DAI guidelines/code of practice are used if appropriate**

*SPIRIT example: “*All activities will be conducted in accordance with the guidelines of the International Association of Human-Animal Interaction Organizations (IAHAIO) to ensure human and animal welfare.” [45].

*CONSORT example:* “The leadership and staff of the program met the previously described standards suggested by the IAHAIO [46] for AAT.” [47].

*Explanation:* The use of established guidelines or codes of practice in DAIs helps ensure the intervention is conducted ethically and safely for all parties involved. Specifying any relevant guidelines/codes of practice used provides transparency and demonstrates a commitment to meeting professional and welfare standards.

### SPIRIT 3d: Composition, roles, and responsibilities of the coordinating site, steering committee, endpoint adjudication committee, data management team, and other individuals or groups overseeing the trial, if applicable.

### CONSORT 5b: Financial and other conflicts of interest of the manuscript authors

- ➢** Extension for DAI trials: Name, accreditation status, non-/for-profit status of any AAI organisations involved in intervention (if applicable).**

While the first SPIRIT example provided by Walker et al. [48] addresses most of the important aspects of this extension item, it does not include the accreditation status. As such, we provide two SPIRIT examples which provide comprehensive cover of this extension.

*SPIRIT example 1:* “Participants select activities to engage in with the dog from a 22-item list curated by Pet Partners, an animal-assisted therapy national organisation. The activities are specifically designed by the non-profit organisation with the goal of helping individuals maintain health or regain their full potential with AAI, and include activities such as petting the dog, talking to the dog, teaching the dog tricks and brushing the dog.” [48].

*SPIRIT example 2:* “All therapists following this treatment protocol will ensure they meet their legal obligations under the Animal Care and Protection Act 2001 and Australian Code for the Care and use of Animals for Scientific Purposes 2013.” [43].

The example provided below for the CONSORT extension could be improved by stating the not-for-profit status of the service dog foundation.

*CONSORT example: “*The AAT program for this trial was developed by therapists and dog behavioural specialists from the Dutch service dog foundation Stichting Hulphond Nederland and psychologists from the mental health care organization GGZ Oost Brabant who have a specialization in autism. […] The Dutch service dog foundation Stichting Hulphond Nederland provided the therapy dogs.” [49].

*Explanation:* The accreditation status of the organisation demonstrates its adherence to recognised standards in the field of Animal-Assisted Interventions, ensuring that the intervention aligns with best practices in both human therapy and dog welfare. Accreditation serves as evidence of the organisation’s commitment to quality and professionalism.

The non-/for-profit status descriptor helps stakeholders understand the organisation’s motivations and financial structure. For instance, non-profit organisations may prioritise community service and accessibility. For-profit organisations might have commercial interests, requiring scrutiny to ensure that participant and dog welfare are prioritised over profit.

### SPIRIT 9a: Scientific background and rationale, including summary of relevant studies (published and unpublished) examining benefits and harms for each intervention.

### CONSORT 6: Scientific background and rationale

The following two DAI extensions apply to these SPIRIT and CONSORT items.

- ➢** Extension for DAI trials: Scientific background and rationale for including a DAI.**

*SPIRIT example:* “Animals have been shown to be highly motivating and to help build the therapeutic alliance in the context of animal-assisted therapy. They can activate implicit motives and intrinsic motivation in humans. This implies that animals can enhance the intrinsic motivation for active participation in psychotherapy. For some people, it may be simpler to establish contact with a person in the presence of a dog; in such cases, the animal acts as a “social catalyst”. Moreover, people can be perceived as more trustworthy by others when they are accompanied by an animal. The presence of an animal may thus positively affect the therapeutic alliance in psychotherapy […] To our knowledge, no study on animal-assisted therapy has investigated the therapeutic alliance and motivation in psychotherapy for children and adolescents as the primary outcome, even though these factors are known to be key elements for successful psychotherapy. In addition, there are no consistent data on how the way a dog is integrated into a psychotherapeutic setting affects outcomes.” [45].

*CONSORT example:* “We considered the effects of AAT in children with ASD [50, 51] and hypothesised that in adults with ASD, AAT may result in stress reduction, improvements in social responsiveness (social awareness, communication, and motivation), and reduced depressive and anxiety symptoms, which are strongly related to stress [52]. Considering that stress-reducing effects of dogs were reported in the general population [53] and that dogs are the animals most commonly employed in AAT with children with ASD, [54] our aim was to explore the effects of AAT with dogs in adults with ASD with normal to high intelligence. We focused on self-perceived stress, social responsiveness, and psychological symptoms (such as depression and anxiety symptoms). Furthermore, we looked at AAT’s effects on self-esteem in this same group—an important addition, given that adults with ASD were found to have lower self-esteem than adults without ASD and that self-esteem is strongly negatively correlated with the stress-related outcomes of depression and anxiety [55].” [56].

*Explanation:* Providing a scientific background and rationale for including a DAI as the intervention of choice ensures the intervention is grounded in evidence, aligns with the research objectives, and addresses the needs of the target population. By outlining the empirical support for DAIs specifically, this extension promotes the credibility of the research and helps provide a context for the work.

- ➢** Extension for DAI trials: Description of proposed mechanism(s), model(s) or theories describing the potential impact of the DAI, if applicable.**

*SPIRIT example:* “Mechanisms explaining the effects of animals on prosocial behaviour vary from a social catalyst effect of animals on humans, to a social support effect of dogs on humans. Animals can provide a comforting and supporting environment, where humans can experience a consoling place to talk and touch. Another theory is that animals have a modelling effect on humans. They provide immediate feedback, which may help humans learn appropriate social behaviour including the relationship between cause and effect. Through role assumption, people are able to fulfil the role of teacher or caretaker in a human-animal interaction and may therefore display more nurturing behaviour [57–60]. On a physiological level, increases in prosocial behaviour are explained by, for example, an increase in oxytocin resulting from human-animal interaction [53, 61, 62].” [63].

*CONSORT example: “*It has been hypothesized that touching the animal reduces stress [64] and expression of non-verbal communication (body posture, gestures) improves social communication [39]. Therefore, the therapy protocol contained several exercises aiming to reduce stress and improve social communication.” [65].

*Explanation:* Information relating to potential underlying mechanisms is crucial for advancing scientific understanding of DAIs. It supports hypothesis development, informs intervention design and the selection of outcome measures and helps readers understand why the intervention is expected to have specific effects. For example, a potential mechanism might involve the release of the hormone oxytocin in humans when interacting with dogs, which is associated with stress reduction and increased social bonding [66]. Describing potential mechanisms helps to link the intervention to relevant biological, psychological, or social theories. Detailed descriptions or modelling of hypothesised mechanisms can inform adaptations of the intervention to different populations or settings, guide the design of future studies to focus on those elements, potentially leading to more refined or targeted interventions [67]. Researchers should also be aware that mechanistic processes are often complex, multiple, and rarely operate independently. Where possible, authors should avoid or at least acknowledge oversimplifications as this risks a reductionist approach.

### SPIRIT 10: Specific objectives related to benefits and harms

### CONSORT 7: Specific objectives related to benefits and harms.

- ➢ **Extension for DAI trials: Specific objectives or hypotheses related to the DAI**

*SPIRIT example:* “Aim 1: To determine if including a therapy dog into occupational therapy sessions can increase the amount of time engaged in on-task behaviour within the session for children with ASD.

Hypothesis 1: Children on the autism spectrum who participate in canine-assisted occupational therapy will spend more time actively attending to a task compared to children receiving usual care occupational therapy.” [43].

*CONSORT example:* “The aim of the present study was to examine the effect of the treatment rationale on pain in an AAI […] We hypothesized that DT [Dog Treatment] and PT [Placebo Treatment] would lead to increased heat-pain tolerance and to decreased self-reported ratings of unpleasantness and the intensity at the limit of participants’ heat-pain tolerance at post-treatment compared to no treatment (primary hypothesis). As secondary hypotheses we assumed the post-treatment heat-pain threshold, the intensity at the heat-pain threshold, the expectations of pain unpleasantness, and the intensity at the limit of tolerance after the treatment to be lower, and the trustworthiness of the investigator to be higher in the DT and PT groups compared to NT [No Treatment]” [68].

*Explanation:* DAIs are often used as an adjunct to therapeutic/interventional research to facilitate the impact of intervention [69–71]. However, DAIs may also be used as a standalone intervention [72]. Specifying the objectives and hypotheses increases clarity and informs the study's design, including the selection of appropriate methods, tools, and participant criteria. For instance, if a hypothesis proposes that DAIs reduce stress in participants, the design should incorporate stress measurement tools.

### SPIRIT 13: Settings (for example, community, hospital) and locations (for example, countries, sites) where the trial will be conducted.

### CONSORT 11: Settings (e.g., community, hospital) and locations (e.g., countries, sites) where the trial was conducted.

The following three DAI extensions apply to these SPIRIT and CONSORT items.

- ➢** Extension for DAI trials: Description of how considerations related to dog welfare are incorporated in the study context and settings.**

*SPIRIT example: “*To ensure animals’ wellbeing, dogs will be limited to 4 h/week and a rest of more than 15 min between sessions when there are two consecutive sessions. Furthermore, dog-training sessions must not be more frequent than 4/day and must last less than 10 min each one. The dog must not be hungry when training and must always have access to fresh water. All these statements have been followed in other studies [73, 74] and will be supervised by the head and academics of the Animal-Assisted Intervention Office of the University.” [75].

*CONSORT example:* “Therapy dog welfare and signs of distress were monitored during each session by a trained research assistant, familiar with the therapy dogs. Therapy dogs were provided with a 15-min opportunity to settle prior to the session commencing, during which the research assistant would monitor their body language and behaviour. When the dogs were present on camera, they were seated on the floor or a large sofa with their handler, much like they would be at home. When dogs were not present in the video, they were provided with a comfortable spot to rest close to their handler (but out of camera view) and were under the supervision of a volunteer with whom they were familiar. The total involvement of the dogs was a maximum of 15 min in front of the camera and one-hour in total on-location. In addition, the dogs were only permitted to be involved in sessions twice per week and not on consecutive days; typically, they were involved only once per week. If a dog (or handler) showed any signs of distress during a filming or Zoom session, they were sent home; this did not occur in this study, and no incidents of canine distress were reported.” [76].

*Explanation:* Providing transparent information on the measures taken to ensure dog welfare reassures the public that the research is conducted responsibly and ethically, fostering greater trust in the study’s findings and the researchers involved. Many institutions, ethics committees, and journals require evidence that dog welfare standards are being met. Including detailed protocols for dog care aligns with these ethical mandates and promotes humane research practices.

- ➢ **Extension for DAI trials: Description of the characteristics of study settings and the DAI that may affect dogs (e.g., room size, noises, smells, lighting).**

*SPIRIT example:* “The dog’s environment is monitored to ensure it is comfortable for the dog (e.g., temperature, clutter). The therapist is to ensure the dogs are provided with a break every one to two hours depending on their previous workload. This break is to be a minimum of ten minutes outside […] The therapist will ensure that a clean mat and bowl of water is placed safely in the room (away from places the water can easily be spilt). The mat is to be used as a'safe zone'if the dog appears uncomfortable and is needing space. The client is not allowed to physically enter this space during this time […] The dog will be worked off lead at all times or on loose flat collar if required (e.g., if outside) allowing the dog the opportunity to move away if uncomfortable.” [43].

*CONSORT example:* “It must be noted, however, that the effect of room temperature on panting was probably substantial in this study because the temperature is maintained relatively high (22 ± 1) during postoperative awakening and remained constant between evaluations. No prior activity or stimulation was performed that could have influenced panting in the dog. The dog did not show noticeable signs of distress: she spent most of her time oriented to the environment or being passive. She never showed any withdrawal behaviour and interacted both with the child and other people present in the room. The dog explored the environment especially during the early AAT sessions.” [77].

*Explanation:* Dogs possess highly developed sensory abilities. All characteristics of the study setting can influence a dog’s welfare, behaviour, and ability to work in a DAI [78–81]. Factors such as sensory input and physical attributes of the environment (e.g., noise, smells, lighting, type of flooring, space, and temperature) may either support or hinder the dog’s comfort and engagement. Consideration of the sensory environment may be important for dog selection; different dogs may respond differently to sensory inputs. A clear description of these characteristics ensures the setting is appropriately evaluated, adjusted and reproducible. It is important to note that the examples provided are illustrative rather than prescriptive. They serve to demonstrate good practice but many not apply universally across all dog breeds, temperaments, or contexts. What is considered a comfortable or appropriate setting for one dog (e.g., a Labrador Retriever) may not be suitable for another (e.g., a brachycephalic breed such as a Bulldog).

- ➢ **Extension for DAI trials: Description of how the suitability of the dog for the DAI in the given study context and environment is ensured.**

*SPIRIT example:* “The dog will be well socialised to people of all ages, and have completed therapy dog training and assessment for temperament and behaviour, including: friendly, calm and predictable reactions to children, medical equipment (e.g., crutches), client infirmities (e.g., uneven gate, stimming), loud noises, other animals, a range of touches including hugs, items being thrown, toys, and fast movements (e.g., children running); comfortable in a range of settings; emotionally mature; drawn to people, and; confident [82–85]. […] The dog will act calmly and predictably when working off lead in a contained environment, such as a clinic room [82–85]” [43].

*CONSORT example: “*The dogs were then examined by the Committee, who assessed their health, their behaviour when placed in contact with humans and their ability and suitability (in terms of obedience) for involvement in the study. Then the selected dogs were submitted to specific training with their trainers (EV, MP, TP, VC) in compliance with the Institutional Care and Use of Animals Committee of Molina Foundation and of European Institute of Psychology.” [86].

*Explanation:* Ensuring the suitability of the dog involves various assessments (e.g., physical health, personality/temperament, behaviour, training) and helps maximise opportunities for the dog to thrive in the study context and environment, [81, 87] engaging and interacting well with human participants, and performing required tasks without experiencing distress. This process optimises potential intervention benefits and ensures safety for all parties involved. It is acknowledged that different types of DAIs may require different levels of training and preparation. For example, structured therapy interventions necessitate formal handler and dog training, while volunteer-based visiting programmes may follow less intensive but still important welfare-oriented protocols. Regardless of the setting, ensuring the wellbeing and safety of the dog, participant, and handler remains important.

### SPIRIT 14a: Eligibility criteria for participants

### CONSORT 12a: Eligibility criteria for participants

- ➢ **Extension for DAI trials: Eligibility criteria relevant to engaging/interacting with dog, if any (e.g., dog phobia, allergies, history of dog abuse).**

*SPIRIT example:* “Children and adolescents who meet any of the following criteria cannot participate in the study:

- Exhibits acute psychosis or has early childhood autism;
- Is afraid of dogs;
- Exhibits an allergic reaction to dogs;
- Has behaved aggressively toward dogs.

[…] The study team will then check for the inclusion and exclusion criteria and ask the family canine-related questions (concerning their fear of dogs, allergic reactions to dogs, previous experience with dogs, and attitude toward dogs).” [45].

*CONSORT example:* “The exclusion criteria were as follows: (1) severe cognitive impairment, such as aphasia, or inability to follow three-step instructions, (2) allergies to animals, (3) history of asthma, (4) confirmed diagnosis of coagulation disorders, (5) presence of symptoms of dog-related phobia, anxiety, or obsessive–compulsive disorder, and (6) participation in other clinical trials in the past 6 months.” [88].

*Explanation:* Considering factors such as phobias, allergies, or history of dog abuse when identifying potentially eligible study participants is an important clinical and ethical foundation of DAI research and should be tailored to individual study contexts. It can prevent fear/distress, allergic reactions and negative interactions between dogs and participants. To support transparency, it is also important to specify how this information is obtained (e.g., whether through screening tools, medical or history assessments, or other means).

### SPIRIT 14b: If applicable, eligibility criteria for sites and for individuals who will deliver the interventions (for example, surgeons, physiotherapists).

### CONSORT 12b: If applicable, eligibility criteria for sites and for individuals delivering the interventions (e.g., surgeons, physiotherapists).

The following three DAI extensions apply to these SPIRIT and CONSORT items.

- ➢ **Extension for DAI trials: Description of any criteria for sites to ensure dog welfare requirements can be met**

*SPIRIT example:* “The therapist will ensure that the transport of the dog to and from the clinic is safe and comfortable [89]. The dog must not display any form of car sickness. The dog will be worked off lead at all times or on loose flat collar if required (e.g., if outside) allowing the dog the opportunity to move away if uncomfortable [82, 89]. […] The therapist will ensure that a clean mat and bowl of water is placed safely in the room (away from places the water can easily be spilt) [84]. The mat is to be used as a “safe zone” if the dog appears uncomfortable and is needing space. The client is not allowed to physically enter this space during this time. The therapist is to ensure the dogs are provided with a break every one-to-two hours depending on their previous workload. This break is to be a minimum of ten minutes outside allowing the dog to sniff, play, urinate and defecate if needed….The dog’s environment is monitored to ensure it is comfortable for the dog (e.g., temperature, clutter).” [43].

Whilst the CONSORT example provided is less detailed, we recognise that journals often impose strict word limits. As such, we recommend that authors provide a short description of the suitability of the site and refer to their protocol for further details.

*CONSORT example:* “This day ward was well suited to conduct our study, because we had access to many patients with anxiety disorders. Moreover, green and calm surroundings of the ward allow for comfortable and undisturbed contact of subjects with the serving dog.” [90].

*Explanation:* Site-specific requirements address factors related to the needs, safety and comfort of the dog, minimising the potential risk or harm to the dog and others. The description of clear criteria or standard processes demonstrates a commitment to high ethical standards and highlights the importance of creating an environment that prioritises the health, safety, and comfort of the dog(s). This ensures that all sites, regardless of location, operate under the same welfare principles, minimising risks associated with a stressed dog and maximising the potential for the DAI to be effective, thus avoiding disparities in treatment.

- ➢** Extension for DAI trials: Selection criteria for the dog(s) involved in the intervention and justification for these.**

*SPIRIT example:* “The dog participating within this program will be over twelve months of age [82, 85, 91]. The dog will have local council registration. The dog will be well socialised to people of all ages, and have completed therapy dog training and assessment for temperament and behaviour, including: friendly, calm and predictable reactions to children, medical equipment (e.g., crutches), client infirmities (e.g., uneven gate, stimming), loud noises, other animals, a range of touches including hugs, items being thrown, toys, and fast movements (e.g., children running); comfortable in a range of settings; emotionally mature; drawn to people, and; confident [82–85]. Re-assessments of the included dog will occur every twelve months unless concerns are noted earlier by the therapist. The dog must complete a bi-annual, full veterinarian health check to ensure physical and emotional health, and confirming vaccinations as well as, have regular treatments for fleas, ticks and parasites [82–85]. The dog will be able to complete the following tasks and commands consistently under distractions and without food rewards: walk on loose lead through a crowd of people, sit, drop and stay, recall, leave it, on your mat, accept a friendly stranger and sit politely for patting [82–85]. The dog will act calmly and predictably when working off lead in a contained environment, such as a clinic room [82–85]” [43].

*CONSORT example:* “The selection criteria for the therapeutic dog were as follows: (i) free of all veterinary infectious diseases; (ii) no abnormal findings in comprehensive health check-ups; (iii) completion of all necessary vaccinations; (iv) moderate grades on aptitude and technical tests; and (v) participation in previous therapy programmes. The pet partners handled preparation and management of the therapy dogs, which were bathed and sanitized at least 24 h before the programme.” [92].

*Explanation:* Describing factors such as demographics (e.g., breed, age, sex), personality, behaviour, training, and health status supports transparency and replicability. Some intervention contexts may require specific traits (e.g., calm, social dogs for therapeutic settings). Consideration of this ensures individual dogs are suited for the tasks, minimising risk of harm or adverse effects. Where relevant, authors should also consider describing the working relationship between the dog and the handler/therapist, as this relationship may impact responsiveness and safety of interactions during the sessions.

- ➢** Extension for DAI trials: Description of relevant training and education undertaken by all parties that is pertinent to their role in the intervention (e.g., participant vs. dog handler). This may include training on dog welfare, safety and intervention-specific content.**

*SPIRIT example:* “The therapist must have completed additional handler training (minimum five-day, in person, handler-canine training) allowing safe and ethical practice. Topics included within this training program would include what is AAT, impact of human-animal interaction, client and animal ethics, animal well-being (including zoonosis), documentation and reporting, policies and procedures, insurance, canine body language, interventions, and current research.” [43].

*CONSORT example*: “The therapists providing AAT had a college or university degree in mental health care and were specialized in working with adults with ASD. Additionally, the AAT therapists had completed advanced courses in dog behaviour and welfare.” [93].

*Explanation:* Describing training and education ensures the individuals possess necessary skills, experience, and qualifications to facilitate safe and effective interventions. These criteria support the wellbeing of all parties involved and promote ethical practices. Transparency in these criteria helps to standardise DAI practice and facilitates replication across studies.

### SPIRIT 15a: Intervention and comparator with sufficient details to allow replication including how, when, and by whom they will be administered. If relevant, where additional materials describing the intervention and comparator (for example, intervention manual) can be accessed.

### CONSORT 13: Intervention and comparator with sufficient details to allow replication. If relevant, where additional materials describing the intervention and comparator (e.g., intervention manual) can be accessed.

The following five DAI extensions apply to these SPIRIT and CONSORT items.

- ➢** Extension for DAI trials: Description of DAI goal and content.**

*SPIRIT example:* “The therapy sessions will be structured in accordance with the individual therapeutic goals of each patient.

Overview of the three conditions:

1. Therapy sessions with a psychotherapist and the mere presence of a dog that is not integrated into the therapeutic narrative. The dog will be present in the therapy room and can interact with the child/adolescent, but it will not be specifically involved in the therapeutic process until the follow-up questionnaires are completed. The children and adolescents will be told that the dog is present because it would otherwise be alone at home.
2. Therapy sessions with a psychotherapist and a dog that is part of the therapeutic narrative and included in the therapeutic process until the follow-up questionnaires are completed. In this condition, the children and adolescents will be told that the dog is present because it is a therapy dog and that it is there to support the child or adolescent in difficult situations. The dog will thus be part of the therapeutic context. The psychotherapists will receive a manual with ideas for how to actively involve the dog in the sessions.
3. Therapy sessions with a psychotherapist but without a dog. A dog will not be present in the therapy room until the follow-up questionnaires are completed.” [45]

*CONSORT example: “*The primary goal of the AAT was to improve the negative symptoms (blunted affect, emotional withdrawal, social withdrawal, lack of spontaneity, and flow of conversation) and general psychopathology symptoms (anxiety, depression, uncooperativeness, disorientation, and poor attention) of patients. Secondary goals were aimed at improving positive symptoms (conceptual disorganization, suspiciousness, persecution, and hostility) and patient well-being. The AAT sessions were conduct[ed] in a spacious and quiet classroom, with the participants seated in a semicircle. The animal assisted therapist, occupational therapist, and therapy dog were positioned in front of the participants. The dog approached the participants in turn, and each participant walked the dog around the classroom. Each AAT session was carried out according to a similar overall structure: 15-min warm-up, 45-min therapeutic activities, and 5-min feedback. In the warm-up, the animal assisted therapist started by greeting each participant, introduced the therapy dog, reviewed the contents of the last session, and oriented participants to the therapeutic activities. There were four types of therapeutic activities carried out to achieve the therapeutic goal: activity for positive emotion, social activity, cognitive activity, and physical activity. Activities aimed at positive emotion included touching the dog, singing a song, massaging the dog, playing with the dog (ball, loop, game), and artistic creation (dot art). Social activity involved introducing, greeting, praising, thanking, helping, talking, making appropriate physical and eye contact, and cooperating in the games with each other and the dog. Cognitive activity included questions and answers, training the dog, orienting the content of activity, playing a cognitive game (puzzle, triangle, and memory card), and writing a worksheet. Physical activity involved walking, handling, feeding, grooming, dressing, and doing exercises with the dog. Each activity was performed for three sessions with gradually increasing levels of difficulty. Therapists gave feedback on what the group did during the therapeutic activities, asked how they felt with the dog, and previewed the content of the next session.” [94].

*Explanation:* Defining the intervention goal ensures that all aspects of the study, including methodology and analysis, align with the intended purpose. A well-defined goal, where applicable, enables the identification of specific, measurable outcomes that can be assessed to evaluate the intervention's impact and identify extraneous variables to control for. Describing the content helps to ensure that the intervention is delivered consistently, supporting replicability.

- ➢** Extension for DAI trials: Description of the proposed duration and frequency of DAI including, where possible, justification.**

*SPIRIT example*: “The presented AAT program will compromise [comprise] 20 sessions in 10 weeks […] Participants will receive the sessions twice per week and the therapies will always be conducted at the same time […] Each session will last 30 min. The structure of each will follow the following order. First, a welcome period of 5 min, during which participants and technicians will introduce themselves with greetings between the patients and the dogs. Secondly, the central part of the sessions, with a duration of 20 min, will be focused on the specific objectives of the therapy. It will consist of the execution of diverse orders given by the professional, such as performing a circuit that includes active listening, going up and downstairs, ambulation, manipulation of objects and activities that involve interaction with their companions and with the dog. The sessions will end with a farewell period of 5 min in order to say goodbye to the dog, sometimes with food, sometimes only verbally, gesturally, and/or with petting. This approach has been followed by previous studies. [95, 96]” [75].

*CONSORT example:* “Participants were offered a total of nine, weekly, one-hour, animal assisted occupational therapy sessions. This was based on clinical experience as no existing protocols, indicating the optimal number of sessions, were available.” [97].

*Explanation:* Duration and frequency of the intervention are core parameters that are likely to affect its impact. For example, too short or infrequent sessions may not produce measurable effects, while overly frequent sessions could cause stress to the dogs or fatigue for participants. Providing the justification, where possible, for these parameters helps to demonstrate that the proposed duration and frequency are appropriate for achieving the intervention goal(s) while considering the welfare of the participants and the dog. This information also allows researchers to evaluate the impact of exposure to the intervention and supports replicability.

- ➢ **Extension for DAI trials: Description of tasks and roles of each individual in the DAI team (i.e., dog, dog handler, other trained professional) including details of participant-dog interactions.**

*SPIRIT example:* “Dog handlers’ responsibilities are: (a) to be responsible at all times for the dog’s interaction; (b) to identify him/herself and the dog before accessing at the hospital; (c) to ensure dog hygiene and well-being conditions; and (d) to provide necessary material for the development of the activity.” [74].

*CONSORT example:* “A participating dog was required to be in the room for all sessions where the youth was engaged in a portion of treatment, including the conjoint session with the caregiver […] The clinician provided cues for the dog to approach or move away in accordance with the youth’s preferences. During situations where the youth appeared stressed, the clinician offered interaction with the dog as a coping skill alongside previously taught skills (e.g., relaxation), but did not otherwise introduce the dog into the activities of the sessions. The dogs’ handlers visually observed all treatment sessions from behind a one-way mirror in an adjacent room to ensure the safety of the animals. Audio was turned off to protect the confidentiality of participants. Handlers were free to stop sessions at any point by knocking on the treatment room door if they were concerned for the dog’s welfare; only one such instance occurred. Five separate dogs participated in the trial, all of which were Labrador retrievers bred and trained to work as service dogs. When a youth was randomized to the TF-CBT AAT [Trauma Focused-Cognitive Behaviour Therapy Animal Assisted Therapy] condition, the assigned clinician scheduled the appointment with the caregiver at a predetermined time that a dog would be available and then notified the lead handler. The lead handler selected the specific dog/handler team that would be assigned for the duration of the youth’s participation in the trial. The assigned dog was considered an extension of the assigned clinician; if for any reason the dog was unable to attend (e.g., sickness) it was considered the same as the clinician being unavailable and the session was re-scheduled.” [69].

*Explanation:* Explicitly stating roles and tasks increases transparency and replicability, acknowledges the potential complexity of intervention dynamics and reduces the risk of unaccounted-for influences (e.g., differing levels of handler expertise or inconsistent participant-dog interactions). Dog handlers may have a dual role (e.g., simultaneously clinician and dog-handler), [69, 71] or single role (e.g., handling the dog in the intervention setting, alongside potential other professionals/clinicians) [33].

Including specific details on expected participant-dog interactions highlights how the intervention goal(s) are expected to be achieved. These interactions may be passive and/or low intensity (e.g., dog is present with no direct engagement) [98] or active and/or higher intensity (e.g., brushing or petting the dog) [86, 88, 99–101]. Dogs can also be involved in either a passive or active way depending on the needs and goals of the participants [43, 102].

- ➢ **Extension for DAI trials: Where possible, consideration of suitability/tailoring of DAI under EDI aspects (e.g., severity of illness).**

*SPIRIT example:* “Specific strategies have been identified to support the engagement of children on the autism spectrum. Beginning the session by incorporating the child’s special interests has been posited to provide a more playful environment in which the child is more likely to engage. Incorporating the use of visuals supports, such as activity schedules and social stories, has been shown to enhance the child’s sense of autonomy, whilst reducing the need for adult prompting.” [43].

*CONSORT example:* “The programme was designed to be as inclusive as possible, and the different activities were conceived to be simple and flexible in order to adapt to each participant's needs and abilities. The team was allowed to make simple changes so each participant could complete all activities (i.e., for participants having serious motor disabilities, activities in session 13–15 were simplified).” [103].

*Explanation:* Addressing equality, diversity, and inclusivity in the design and implementation of a DAI helps maximise opportunities for the intervention to be accessible, appropriate and beneficial to all participants involved. People from different backgrounds may have varying perceptions and experiences with dogs. For example, dogs may be viewed as unclean or dangerous by some, [104] while others see dogs as companions. Understanding these differences potentially helps to avoid discomfort or distress in participants, ensuring that the intervention is appropriate and acceptable to them. Identifying individual perceptions can inform the introduction/familiarisation processes and the types of interactions involved in the intervention. The authors note that some countries can have different laws around EDI data collection which may influence the applicability of this extension.

- ➢ **Extension for DAI trials: Rationale for and description of the comparator group.**

**Fig. 1:**
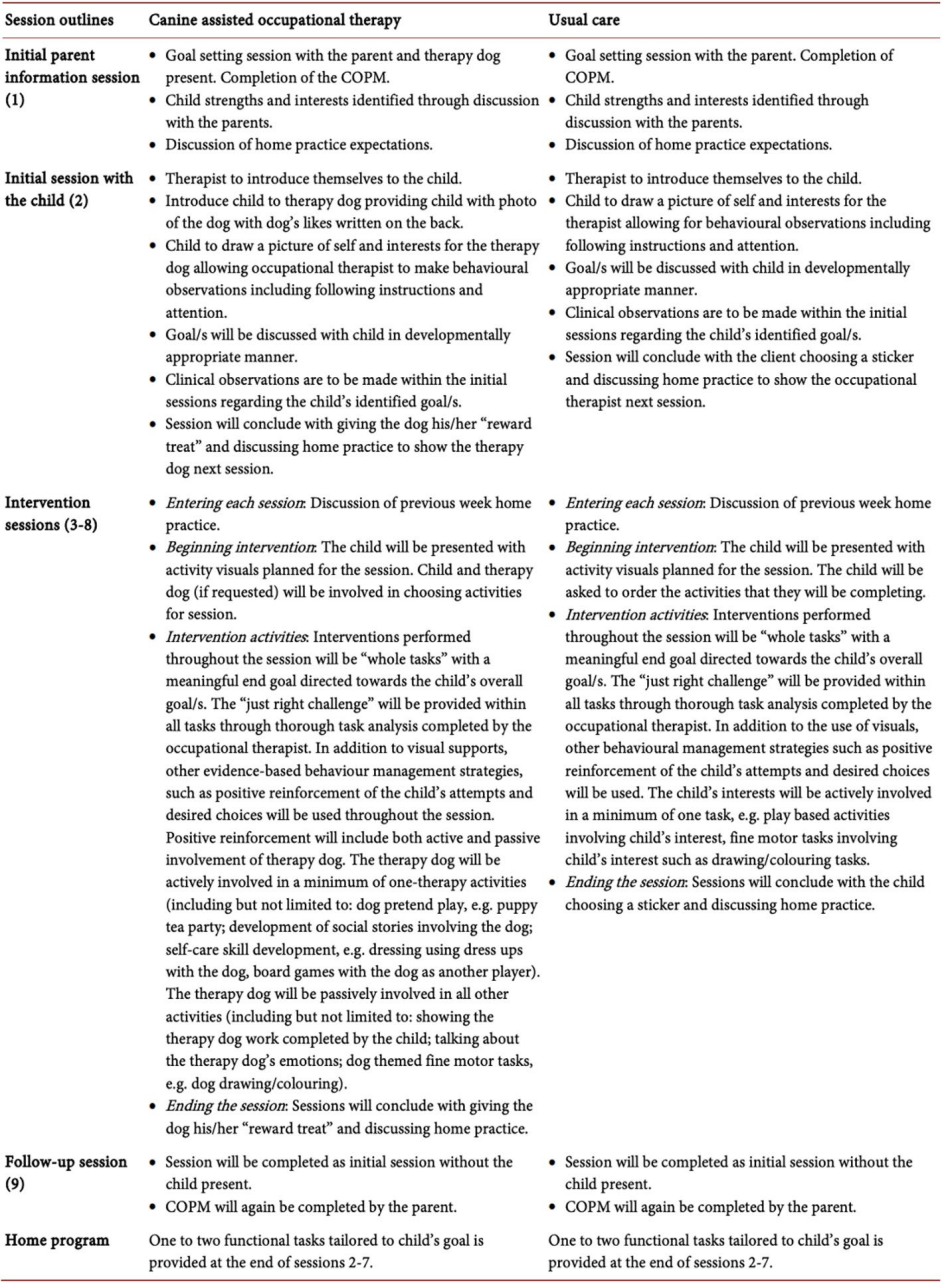
Table illustrating good reporting for SPIRIT item 15a, DAI extension: Rationale for and description of the comparator group

*SPIRIT example:* “The usual care control group will follow a similar structure to the treatment group (Table 3, presented here as Fig 1). A total of nine, weekly, one-hour sessions will be completed by the first author without her therapy dog present. Sessions will be conducted within a therapy clinic. An in-depth manual will be developed allowing for replication (Table 3, presented here as Fig. 1). Within the initial and final sessions, the COPM [50] will be also completed. Child sessions will involve evidence-based interventions without the inclusion of the dog. Strategies used to encourage intrinsic motivation to engage will be as indicated within the treatment group with the exclusion of the therapy dog [105–107]. At the completion of the trial, participants within the usual care control group will be offered a block of eight animal assisted occupational therapy sessions. This will be offered to ensure equity amongst participants and no data will be recorded during this period.” [43].

*CONSORT example:* “Each patient in the control group was assigned to a single activity from the functional program on the basis of their therapist’s criteria but taking into account the individual’s preferences. The choice was between art therapy, group sports (football or basketball), dynamic psycho-stimulation, and gymnastics. These activities were organized so that they closely matched certain important characteristics of the AAT program:

- They were conducted outside the hospital unit where the patients were resident.
- They all involved a similar element of group work.
- Group sizes were small (similar to the AAT sessions).
- Patients were accompanied and supervised off-site by a mental health professional (nurse or similar).
- The activities continued throughout the period of trial (they were unaffected by season).
- The sessions were twice-weekly and of 1-h duration.

The difference between functional program activities and the AAT sessions was, as far as was possible, restricted to content.” [99].

*Explanation:* Describing the chosen control group (‘usual care’ or an alternative active treatment, e.g., a robotic animal intervention or intervention involving cuddly toys) including rationale for the choice is important for transparency and to enable quality assessment of the inferences drawn from the study. For DAIs it can be difficult to provide a suitable comparator for the presence of a dog. Therefore, it is necessary to describe transparently the similarity of intervention and any quality control or safety assessments.

### SPIRIT 15b: Criteria for discontinuing or modifying allocated intervention/comparator for a trial participant (for example, drug dose change in response to harms, participant request, or improving/worsening disease)

- ➢ **Extension for DAI trials: Criteria and/or processes for discontinuing or modifying the intervention based on dog and/or handler responses.**

*SPIRIT example:* “The intervention will be modified or ended if any unpredictable or adverse events occur. This could include if the child or adolescent exhibits an allergic reaction, fear, or aggressive behaviour toward the dog. Aggressive behaviour by the dog will also qualify as an adverse event. Adverse events will lead to exclusion from the study.” [45].

*Explanation:* Clearly specifying criteria and/or processes for discontinuing or modifying a DAI ensures the safety, wellbeing, and ethical treatment of all parties involved. Establishing these criteria and/or processes proactively allows for prompt responses to adverse situations, safeguarding the physical and mental health of both humans and dogs while maintaining the integrity of the study. The criteria and processes for discontinuing or modifying the intervention should include the following:

- Step-by-step protocols for pausing, modifying, or ending the session.
- Criteria for rescheduling or adapting the intervention to suit needs.
- Emergency procedures for managing unexpected incidents.

### SPIRIT 15d: Concomitant care that is permitted or prohibited during the trial

- ➢ **Extension for DAI trials: Description of whether and how potential concomitant human-dog interactions (e.g., with pet dog) are controlled for.**

A suitable SPIRIT example was not found. Therefore, we have created this example as an illustration of what authors may wish to consider.

*SPIRIT example: “*Participants will be required to complete a validated scale to assess their bond with their pet dog and the approximated number of hours spent interacting with their dog each week. Participants will be categorised into ‘high’, ‘medium’, or ‘low’ interactors based on their responses to these survey items and used as a variable in sub-group analysis.”

*Explanation:* Uncontrolled interactions with pet dogs or other non-intervention dogs may influence the participant's emotional, behavioural, or physiological responses to the intervention under assessment. For example, a participant with a strong bond with their own pet dog might experience less impact from an intervention dog. Alternatively, a negative or stressful interaction with a pet dog could dampen the participant's ability to engage effectively with the intervention dog. This may reduce the focus on the intervention and can make it harder to isolate its unique contributions. Accounting for concomitant interactions where possible helps contextualise study findings.

### SPIRIT 16: Primary and secondary outcomes, including the specific measurement variable (for example, systolic blood pressure), analysis metric (for example, change from baseline, final value, time to event), method of aggregation (for example, median, proportion), and time point for each outcome.

### CONSORT 14: Prespecified primary and secondary outcomes, including the specific measurement variable (e.g., systolic blood pressure), analysis metric (e.g., change from baseline, final value, time to event), method of aggregation (e.g., median, proportion), and time point for each outcome.

The following two DAI extensions apply to these SPIRIT and CONSORT items.

- ➢ **Extension for DAI trials: Outcome measures relevant to the proposed pathway of action of the DAI.**

*SPIRIT example:* “Incorporating a dog into the learning environments of children is thought to act as an internal motivator, reducing the child’s self-consciousness, increasing attention and awareness of their social environment, as well as, their playfulness […] All therapist and child interactions during the therapy session will be video recorded and a total of 44 (first and last session of all children) will be coded by two non-study personnel for the time spent actively attending to the therapy task, as well as, fidelity of therapist delivered intervention. On-task behaviour is defined as “physical contact with one or more objects in a manner that could result in completion of a task” [[108]: p. 262]. Examples of on-task behaviours within this study may include collecting materials needed to complete the task, manipulating materials in a way that is directed towards task completion and requesting assistance from the therapist.” [43].

*CONSORT example: “*The rational for therapy dogs is supported by prior literature that demonstrates that human perception of stress and pain can be reduced with exposure to animals [109–113]. Other studies have found reduction in stress using therapy dogs in multiple healthcare settings [111, 113]. An average 12-min exposure to a therapy dog reduces anxiety in 34% of fibromyalgia patients, together with reductions in pain and improvements in mood [110]. […]. The study hypothesis was that patients exposed to a therapy dog would have lower perceived anxiety than patients with usual care while in the ED [Emergency Department]. The primary outcome measure was the patient-reported anxiety score (0–10: 0 = balanced mood [least anxiety], 2 = slight fear and worry, 4 = mild fear and worry, 6 = moderate worry, physical agitation, 8 = feeling really bad, at the edge, 10 = out of control behaviour, self-harm [worst anxiety]) […]. These three measurements (anxiety, pain, and depression) were obtained three times: at baseline (T0, prior to dog or control), and then again, about 30 min after exposure to the dog or usual care (T1), and then as late as possible prior to patient discharge (T2). Patients with a 0 score on anxiety were screen failures […]. Physicians, blinded to the patients’ self-assessments, were asked to rate their patients’ anxiety, depression and pain on the same scales as were used by the patient prior to intervention. The dog handlers were asked to provide written field notes with instructions to record their impressions of the patient’s words, affect and behaviours before during and after interaction with the dog. Handlers were encouraged to report without bias, both negative or positive observations, and to report both direct observations of behaviour, and also their own interpretations of the patient’s mood, affect or emotional state.” [114].

*Explanation:* Specifying outcome measures that directly align with the intended effects of the DAI ensures the study evaluates intervention-specific impact. For example, if the DAI aims to reduce anxiety, measures like self-reported anxiety scales or physiological markers (e.g., cortisol levels) should be included. If the goal is to improve social interaction, measures might include frequency and quality of social behaviours. The success of a DAI will, in part, depend on the dog’s behaviour and demeanour (e.g., calmness, attentiveness, responsiveness). Distress or discomfort in the dog can negatively impact their interactions with participants, [115] potentially diminishing the intervention’s therapeutic impact. Active monitoring of the dog enables learning that can lead to protocol improvements, such as session duration, frequency, or type of activities, to better suit the needs of both dogs and participants.

- ➢** Extension for DAI trials: Where applicable, justify the use of unblinded outcome measures in relation to the DAI.**

*SPIRIT example:* “The therapists will be blinded until after the initial meeting with the patient and their family. Afterward, they will need to know if they have to plan the dog for the sessions to plan their schedules with the patients.” [45].

*CONSORT example:* “The laboratory technicians who analysed the saliva samples were only given the patients’ ID numbers and were blinded to whether patients were in the control or AAT-treatment group. For practical reasons and for issues relating to the availability of resources and personnel, the rest of the process of the study could not be blinded. It was not possible for patients to be blinded to the presence of dogs, and only one hospital neuropsychologist was able to participate in the study (in charge of all of the pre-treatment and post-treatment evaluations of the study, and follow-up of all of the patients). A single researcher not only carried out the collection of the data and saliva samples but also acted as a guide for the therapy dogs during the AAT sessions.” [99].

*Explanation:* In unblinded studies, researchers, handlers, or participants may consciously or unconsciously influence the outcome assessments based on their expectations about the intervention's effectiveness. Justifying and clearly reporting unblinded measures enhances the trial’s transparency and reliability.

In some DAIs, it may not be possible to blind participants or assessors due to the visible presence of the dog during the intervention [88, 94, 97, 116–118]. Justifying unblinded measures shows that this limitation has been considered and that alternative strategies are employed to minimise bias. For example:

- Objective measures: Incorporating physiological markers (e.g., heart rate variability, cortisol levels) that are less susceptible to subjective interpretation.
- Standardised protocols: Ensuring that evaluators follow strict guidelines to minimise bias in subjective assessments.
- Independent assessors: Using independent evaluators who are not involved in delivering the intervention.

### SPIRIT 17: How harms are defined and assessed (for example, systematically, non-systematically).

### CONSORT 15: How harms were defined and assessed (e.g. systematically, non-systematically).

- ➢ **Extension for DAI trials: Observations/measures relevant to the impact of the intervention on the dog(s)**

*SPIRIT example:* “In order to assess the wellbeing of the therapy dogs involved within this study, salivary samples will be taken at multiple intervals to assess for changes in cortisol, alpha amylase, and oxytocin levels. The adrenocortical hormone cortisol has been the most commonly referenced biomarker when assessing psychological arousal and is regularly used as a biomarker for animal welfare [83]. An increase in cortisol will occur as a result of the activation of the hypothalamic–pituitary–adrenal axis in response to a stressful stimulus [83]. Although acknowledged as a useful biomarker of animal welfare, several limitations have been identified [83]. Firstly, cortisol levels within dogs are characterised by high intra- and inter-variability, and are impacted by several demographic and environmental factors, therefore, limiting its generalisability [83]. In addition, when using cortisol as a biomarker to assess animal welfare, it should be understood that elevated levels of cortisol can reflect excitement [83]. Because of these limitations, alpha amylase (sAA) will also be assessed. Alpha amylase is a protein released by the parotid gland as a result of psychological or physical stress [119]. During periods of stress an increased level of sAA is released into saliva [119]. Saliva samples to assess sAA will be taken at the same time intervals as the cortisol [119]. Oxytocin is a neuropeptide related to social behaviour, cognition and stress responses in mammals [120]. Oxytocin is often referred to as the “bonding” or “love-hormone” and has been used as a biomarker to assess HAI. It has also recently been posited as a potential biomarker for animal welfare and, therefore, will be the third salivary measure assessed within our study [120, 121]. Experimental samples will be collected from the dog at multiple intervals during the treatment period using SalivaBio Children’s Swabs […] […] Behaviour occurs as a result of dog’s physical and mental state, with abnormal behaviour being a sign of stress or pain [122]. Observational checklists can be an effective method of assessing animal welfare as they are able to provide detailed information on the animals in a non-invasive matter [122]. Therefore, the Pet Assisted Therapy-Welfare Assessment Tool (PAT-WAT) will be completed. [122]*”*” [43].

*CONSORT example*: “A total of 21 experimental samples were collected from the therapy dog at multiple intervals across a seven-week period of canine-assisted occupational therapy (week one, four, and seven) (Tables 1 and 2) using SalivaBio Children’s Swabs (Synthetic) [123, 124]. No food was provided to the therapy dog for 60 min prior to the sample being taken [121, 125]. The swab was placed between the therapy dog’s right mandibular teeth and cheek (Fig. 1) by the first author for a total of 90 s, with her mouth gently held shut to avoid swallowing [121, 125]. Samples were stored within a sample tube and frozen at − 40 °C immediately after extraction, then transferred to a laboratory on ice within a Styrofoam box and frozen at − 80 °C within 24 h. [125]. A total of three baseline samples were taken from the therapy dog after a typical day at home (week one, four, and seven). Samples were taken at 6:30 p.m. on every occasion, one hour after the first author returned home from work, and a minimum of 30 min after interaction with the first author or others. A food reward was provided immediately after the sample was collected. A total of 18 treatment samples were taken from the therapy dog during the first and last sessions of a typical treatment day (on Thursday of week one, four, and seven; Table 3). Samples were taken at three intervals: (1) five minutes prior to beginning the therapy session, following a 15-min rest period in which there was no human interaction, and a minimum of 30 min after transportation to the clinic; (2) 20 min after initial interaction with the child within the therapy session, and; (3) 30 min after the end of the therapy session, a period in which there was no human interaction. See Table 1 for a summary of the collection schedule. The therapy dog was highly tolerant of having items placed in her mouth (e.g., toothbrush and medications) and showed no adverse reactions to the samples being taken. A food reward was provided after the second and final sample of the session had been taken to ensure there was a minimum of 60 min between the time food was given and sample collection [124, 125] […] In addition to the salivary biomarker analyses, a total of six hours of session video were analysed for canine behaviour. Videoing began a minimum of 1 min before the child entered the room and was stopped 1 min after the child exited.” [126].

*Explanation:* Monitoring the impact of the intervention on the dog(s) is important for ethical and welfare reasons as well as minimising risk of harm from a stressed dog. Stress, fatigue, or health concerns should be monitored and addressed immediately. Future research and practice should aim to prevent situations where a stressed or sick dog might inadvertently cause harm or distress to participants [115]. Using validated instruments and procedures provides more objective data on the dog’s physical/emotional wellbeing, however at a minimum the dog’s signalling should be monitored continuously and acted upon without delay. If possible, monitoring procedures could also be extended to include a multi-method approach combining subjective clinical opinion with physiological measures where appropriate (e.g., heart rate, salivary cortisol levels [81]). This proactive approach supports the identification and mitigation of stress or harm and promotes ethical practice.

### SPIRIT 18: Time schedule of enrolment, interventions (including any run-ins and washouts), assessments, and visits for participants. A schematic diagram is highly recommended.

### CONSORT 22a: For each group, the numbers of participants who were randomly assigned, received intended intervention, and were analysed for the primary outcome.

- ➢ **SPIRIT extension for DAI trials: Time schedule of any required participant-dog training/familiarity sessions prior to commencement of the intervention.**
- ➢ **CONSORT extension for DAI trials: Time schedule and completion rates of any required participant-dog training/familiarity sessions prior to commencement of the intervention.**

A suitable SPIRIT example was not found. Therefore, we have created this SPIRIT example as an illustration of what authors may wish to consider.

*SPIRIT example:* “One month prior to intervention sessions, all children will participate in two 45-min safety training sessions. One on understanding dog body language and one on safe behaviour with dogs. Their understanding of this content will be evaluated through a short question and answer session. Children who score less than 70% or provide any answer that could greatly increase risk of harm, are required to re-complete the training […]. Two weeks prior to the intervention commencing, children will be familiarised with dogs in a 30-min, small group session. During this session, the dog handler will provide the children with some information about the dog (e.g., breed, sex, age, likes and dislikes). Children will be encouraged to ask questions and pet the dog, with dog welfare monitored throughout.”

*CONSORT example:* “Prior to intervention sessions, all children took part in safety training on understanding dog body language and safe behaviour with dogs—this included an interactive presentation followed by a question/answer session. Children also took part in a “do’s and don’ts” activity. This took approximately 2 min to do and aimed at setting clear boundaries for behaviour around the dogs during all sessions. In addition to reducing the potential risk of any incidents, this also ensured that children understood the dogs’ welfare needs and the requirement to uphold the dogs’ needs at all times. Children were familiarized with the dogs prior to intervention in order to eliminate potential novelty effects. Familiarization sessions took place in the week preceding intervention, with approximately 30 min exposure to each dog in small groups. Children were introduced to the dog by the handler and given some general information such as breed, sex, age, likes and dislikes. Children were then encouraged to ask questions in order to gain familiarity with each dog. Children were allowed to pet the dog as the dog was led around the group by the handler to greet the children, if the handler and the researcher agreed and if the dog did not indicate any stress signalling.” [127].

*Explanation:* Training to provide education on dog-interactions, and familiarity sessions allow human and dog participants to get to know each other, reducing potential risks, anxiety or hesitation that could interfere with the intervention [128, 129]. They can help participants understand how to interact with the dog appropriately (e.g., handling, cues, body language), fostering positive and productive interactions during the intervention. Items to consider include:

- Duration and frequency of training/education or familiarity sessions.
- Timing relative to the intervention (e.g., how long before the first session).
- Activities or skills covered (e.g., learning cues, practicing interactions, addressing fears).
- Any adjustments for specific participant needs or preferences (e.g., extra sessions for participants with phobias or disabilities).
- How readiness for the intervention may be evaluated for both participants and dogs.

### SPIRIT 19: How sample size was determined, including all assumptions supporting the sample size calculation

### CONSORT 16a: How sample size was determined

- ➢ **Extension for DAI trials: Description of capacity of dog-handler teams required to safely and effectively deliver the intervention.**

Suitable examples were not found. Therefore, we have created SPIRIT and CONSORT examples as illustrations of what authors may wish to consider.

*SPIRIT example:* “The dog-assisted intervention will be delivered to 200 participants on an individual basis, with sessions scheduled weekly. Each session will have a duration of one hour and will be conducted over a six-week period with eight cycles to complete the intervention delivery within 12 months. To ensure the welfare of the dogs, their involvement will be limited to a maximum of three hours per week. Given the duration of the intervention and the requirement for 200 h of intervention per week, a total of 9 unique dog-handler pairs will be necessary to effectively deliver the intervention throughout the full six-week period. To prevent delay due to dog-handler team absence/illness, three further pairs will be trained as back up. Therefore, in total, 12 unique dog-handler pairs will be required.”

*CONSORT example:* “Twelve dog-handler teams were trained for the intervention to ensure that no dog worked more than three hours a week, and intervention delivery was completed within twelve months.”

*Explanation:* Planning adequate capacity for the provision of DAIs is important to ensure the intervention can be delivered reliably and safely, adhering to relevant AAI guidelines (e.g., [40, 46, 130, 131]). It also ensures the intervention is delivered consistently, reducing variability in outcomes (e.g., due to overworked, unwell, or unavailable dog-handler teams) and increases transparency, enabling planning of resource for future research.

Proper estimation helps allocate other resources, such as space, equipment, finances and time, efficiently. Capacity does not just relate to the number of dog-handler teams required to deliver the number of intervention sessions specified, it should also consider items such as:

- Total number of participants and their individual needs.
- Participant-to-dog ratio for safe and effective interaction.
- Number of sessions, their duration, and frequency.
- Maximum hours a dog can work per day/week.
- Time needed for rest and recovery between sessions.
- The number of trained handlers and their schedules.
- Back-up or replacement dog-handler teams in case of unavailability.
- Activities involved (e.g., high-energy tasks may require shorter working periods).
- Behavioural demands on the dog (e.g., interactions with large groups may be more taxing).

### SPIRIT 28b: Explanation of any interim analyses and stopping guidelines, including who will have access to these interim results and make the final decision to terminate the trial

### CONSORT 16b: Explanation of any interim analyses and stopping guidelines

- ➢ **Extension for DAI trials: Justification, and description, of whether interim analyses and stopping guidelines consider dog welfare.**

The below provide examples in relation to stopping guidelines only, examples were not found for interim analysis. We propose that authors include a statement similar to: “Interim analyses were planned (or performed for CONSORT) at the mid-point of the trial to assess harms related to participant and dog outcomes. These results should be (or were for CONSORT) reviewed by an independent data monitoring committee, with expertise in animal welfare.”

*SPIRIT example:* “The intervention will be modified or ended if any unpredictable or adverse events occur. This could include if the child or adolescent exhibits an allergic reaction, fear, or aggressive behaviour toward the dog. Aggressive behaviour by the dog will also qualify as an adverse event. Adverse events will lead to exclusion from the study. The participants will be able to request to discontinue the study at any time without giving reasons. The therapists will be able to offer the participants psychotherapy outside the study, as they would have otherwise planned.” [45].

*CONSORT example: “*Interventions would be stopped if dogs showed any signs of discomfort or being tired, and handlers were free to take their dog outside for a break as they felt appropriate.” [132].

*Explanation:* Interim analyses provide an opportunity to evaluate findings related to the dog’s welfare, enabling early identification of potential welfare concerns. By embedding specific stopping guidelines for dog welfare, researchers can take prompt action to modify or discontinue the intervention, if necessary, thereby safeguarding the physical and emotional health of all parties involved.

Items to consider:

- Reference to guidelines or best practices (e.g., AAI code of practice, welfare standards)
- Specification of data related to dog welfare (where applicable) to be reviewed during interim analyses (e.g., behavioural observations such as signs of stress, fatigue), physiological measures (e.g., cortisol levels), session reports from handlers or welfare monitors.
- Identify who will conduct/review the interim analysis and how frequently they will occur, what study milestones.
- Define specific welfare-related criteria that would trigger modifications or early termination of the intervention (e.g., persistent signs of stress, veterinary findings indicating healthcare concerns).
- Outline the decision-making process for implementing stopping guidelines/actions based on interim analyses.

### SPIRIT 21b: Type of randomization (simple or restricted) and details of any factors for stratification. To reduce predictability of a random sequence, other details of any planned restriction (for example, blocking) should be provided in a separate document that is unavailable to those who enrol participants or assign interventions

### CONSORT 17b: Type of randomisation and details of any restriction (e.g. stratification, blocking and block size)

- ➢ **Extension for DAI trials: Description of whether concomitant interactions with dogs (e.g., interactions with pet/other dogs) is included as a stratification variable.**

A suitable SPIRIT example was not found. Therefore, we have created a SPIRIT example as an illustration of what authors may wish to consider.

*SPIRIT example*: “We will control for the effects of ongoing interactions with pet dogs by using pet ownership as a stratifying variable in a block randomisation method.”

*CONSORT example*: “Two sets of stratified analyses were also conducted: one for individuals who lived with and without dogs at the time of hospitalization and one for women and men. The hypotheses were tested with the interaction of pre-post with the intervention dummy variables.” [133].

*Explanation:* Without considering concomitant dog-related interactions as a stratification variable, the effects of the intervention may be conflated by the effects of participants’ interactions with other dogs, potentially biasing results. Stratification ensures that groups (e.g., intervention and control groups) are balanced in terms of participants’ pre-existing dog interactions, making the results more reliable and generalisable. Stratifying helps to account for this variability and provides more nuanced insights into how DAIs work for different subgroups.

### SPIRIT 21a: Who will generate the random allocation sequence, and the method used

### CONSORT 19: Whether the personnel who enrolled and those who assigned participants to the interventions had access to the random allocation sequence

- ➢ **Extension for DAI trials: If possible, describe the process of matching dog-handler teams (dog & handler) to participants, including the individual/team responsible for this process, where relevant.**

A suitable SPIRIT example was not found. Therefore, we have created a SPIRIT example as an illustration of what authors may wish to consider.

*SPIRIT example:* “The lead handler will select the specific dog/handler team assigned to each participant for the duration of the trial. Where possible, the handler will choose the dog-handler team based upon blinded assessment of individual participant needs. This will be a subjective matching process with no formal criteria.”

While the first example provided below by Wijker et al. [134] addresses most of the important aspects of this extension item, it does not include the individual/team responsible for this process. Therefore, we provide two examples which together provide comprehensive cover of this extension.

*CONSORT example 1:* “The therapy protocol prescribes a fixed match between the therapy dog and participant. If a different therapy dog (e.g., a more playful one) seemed to offer better opportunities for a participant to reach therapy goals, a change was made in the dog–participant match in the sessions.” [134].

*CONSORT example 2:* “The lead handler selected the specific dog/handler team that would be assigned for the duration of the youth’s participation in the trial.” [69].

*Explanation:* Participants may have specific needs (e.g., fear of large dogs, sensitivity to certain behaviours) that could make interactions with certain dogs uncomfortable, counterproductive, or unsafe. A well-described matching process [131, 132] helps prevent such mismatches. For example, for participants with mobility challenges or physical limitations, the choice of dog (size, weight, energy level) and handler (ability to provide support) is critical to ensuring safe interactions. Some participants may exhibit behaviours (e.g., sudden movements, loud noises) that could stress certain dogs. Whilst participant preferences for a specific dog breed should not take precedence over individual needs and practical factors, considering preferences may be appropriate in some cases, and which case this should be clearly stated.

Handlers may have different skill sets and levels of experience. Matching ensures that handlers are paired with participants whose needs they can effectively address, especially in cases involving complex requirements (e.g., working with children, individuals with disabilities, or participants with trauma histories). This helps to standardise participant-dog-handler interactions, reducing variability that could confound study outcomes. Preliminary meetings could be used to evaluate the compatibility of the pairings before full participation in the intervention [129]. Clear description of selection process (e.g., expert opinion or standardised criteria) and identification of responsible selectors helps to manage liability in case of adverse events.

### SPIRIT 24a: Who will be blinded after assignment to interventions (for example, participants, care providers, outcome assessors, data analysts)

### CONSORT 20a: Who was blinded after assignment to interventions (e.g., participants, care providers, outcome assessors, data analysts)

- ➢** Extension for DAI trials: Description and justification of whether trained professional(s) and dog handler(s) are blinded to the outcomes and how.**

*SPIRIT example: “*The therapists will be blinded until after the initial meeting with the patient and their family. Afterward, they will need to know if they have to plan the dog for the sessions to plan their schedules with the patients.” [45].

*CONSORT example: “*All patients were evaluated by a trained psychologist blind to the patient's intervention group at baseline and after the intervention program (patients were asked not to mention details about the therapy sessions and the psychologist was also not supposed to gather information about their intervention group).” [71].

*Explanation:* In DAIs, the interaction between the dog, handler, and participants can be critical to the intervention. Awareness of expected outcomes might unconsciously alter how the handler or professional facilitates these interactions, potentially skewing the intervention's effects. If blinding is impractical (e.g., the handler must observe outcomes for safety reasons), this limitation should be explained and steps to mitigate its effects should be described (e.g., using third party evaluators). Awareness of the primary/secondary outcomes may be distinguished from the session ‘goal(s)’, as an understanding of this could be needed to support the dog handler to work effectively during the session.

### SPIRIT 27b: Methods for any additional analyses (e.g., subgroup and sensitivity analyses).

### CONSORT 28: Any other analyses performed, including subgroup and sensitivity analyses, distinguishing prespecified from post hoc

- ➢ **Extension for DAI trials: Explain whether a subgroup analysis considers dog characteristics (e.g., size)**.

Suitable examples were not found. Therefore, we have created SPIRIT and CONSORT examples as illustrations of what authors may wish to consider.

*SPIRIT example:* “A subgroup analysis will be carried out with respect to dog size (small dogs: ≤ 10 kg, medium dogs: 11-25 kg, large dogs: > 25 kg) to explore whether the intervention’s efficacy varies based on dog size.”

*CONSORT example:* “A subgroup analysis was conducted based on dog characteristics, particularly size (small dogs: ≤ 10 kg, medium dogs: 11-25 kg, large dogs: > 25 kg). The aim was to explore whether the intervention’s efficacy varied by dog’s size, as dog size could influence participant reaction and engagement.”

*Explanation:* Different dog characteristics, such as size, breed or type, may evoke varying reactions from participants. Analysing outcomes by dog characteristics helps determine whether these factors influence participant comfort, engagement, or therapeutic benefits. Collecting this information may help with tailoring future interventions.

### SPIRIT 28a: Composition of data monitoring committee (DMC); summary of its role and reporting structure; statement of whether it is independent from the sponsor and funder; conflicts of interest and reference to where further details about its charter can be found, if not in the protocol. Alternatively, an explanation of why a DMC is not needed

- ➢ **Extension for DAI trials: Summary of the role of the DMC or other relevant committees in relation to monitoring dog welfare**

A suitable SPIRIT example was not found. Therefore, we have created a SPIRIT example as an illustration of what authors may wish to consider.

*SPIRIT example:* “An independent Dog Welfare Monitoring Committee (DWMC) will review dog welfare outcomes at regular intervals throughout the trial, in addition to reported Serious Adverse Events (SAEs). Dog welfare issues of interest will be specified in the DWMC terms of reference and form part of the Stop–Go criteria.”

*Explanation:* Including a summary highlights the accountability and oversight mechanisms built into the study design. These committees are essential for ensuring acceptable ethical practice involving the use of dogs throughout the intervention and provide a structured process for reviewing welfare concerns, guiding responses to adverse events, and promoting adherence to established dog welfare standards. It is important to note that these considerations may fall outside the scope of university or institutional Research Ethics Boards. Researchers, funders, and Ethics Committees should be aware that, even where not formally required, attention to animal welfare remains a core ethical responsibility and best practice in DAI research.

Items to consider:

- Composition of the Committee involved in monitoring dog welfare, including their expertise (e.g., veterinarians, dog behaviourists, handlers, ethics experts, experienced DAI specialists)
- Frequency of monitoring activities (e.g., how often the committee meet, specification of real-time monitoring for urgent concerns/adverse events)
- Type of data reviewed
- Decision-making processes (description of escalation procedures for critical welfare concerns, including immediate removal of dog from the study if necessary).

### SPIRIT 29: Frequency and procedures for monitoring trial conduct. If there is no monitoring, give explanation.

### CONSORT 27: All important harms or unintended effects in each group.

- ➢ **Extension for DAI trials—SPIRIT: Plans for collecting, assessing, reporting and managing harms or unintended effects for the dog(s).**
- ➢** Extension for DAI trials—CONSORT: Harms or unintended effects associated with the DAI, for the dog, and how these were assessed.**

*SPIRIT example*: “A protocol for using Bella in the intervention will be monitored to reduce potential dog stress and/or fatigue. Brelsford et al. [131] have developed the Lincoln Education Assistance Tool (LEAD) to ensure best practice for dog-assisted interventions in schools. Their Dog Care Plan (see Appendix E) will be adhered to and referred to throughout the intervention. Bella will work two days a week for a one-hour session on each day for the duration of 12 weeks.” [133].

*CONSORT example: “*The number of treatment disrupting events that occurred and were attributable to the presence of the dogs were recorded on fidelity checklists (e.g., barking, whining). Of the 157 completed treatment sessions that included a dog, clinicians identified a treatment disrupting event occurring in 10 of them (6.4%). Six of those events were caused by a single dog who often whined and panted after being separated from his handler […] These six events all occurred in the context of the treatment of a single participant, as the dog was removed from further involvement after the case was completed.” [69].

*Explanation:* Harms or unintended effects may include distress, fear, frustration, fatigue or physical injury resulting from participation. Transparent and systematic approaches to monitoring and responding to these issues ensures acceptable ethical practice. Plans for reporting harms should include the method of assessment (e.g., observations/self-reports from handlers/team members, veterinary assessments, incident reports) and how this should be managed (e.g., immediate actions such as providing rest periods, veterinary care; intervention adjustments; removal from study).

### CONSORT 22b: For each group, losses and exclusions after randomisation, together with reasons.

The following two DAI extensions apply to this CONSORT item.

- ➢ **Extension for DAI trials: Participant losses/exclusions for reasons relating to interacting with a dog.**

*CONSORT example:* “During the program, two patients within the AAT-treatment group withdrew from the study. One patient was discharged from the hospital before the end of the AAT program. The other patient exhibited behaviours that threatened to compromise the welfare of the therapy dogs and therefore stopped participating in the AAT activity.” [99].

*Explanation:* The exact reasons for excluding participants or participant withdrawal post-randomisation should always be clearly reported. It is important these reasons, where possible, consider aspects related to interaction with the dog(s) (e.g., allergies, relationship breakdown, behavioural challenges), and whether losses/exclusions were initiated by the participant or the trial team. Reporting losses/exclusions relating specifically to the dog is important to identify potential barriers to implementation in practice and implications for reliability and generalisability of the results. It is recommended to include this information in the CONSORT flow diagram.

*CONSORT example:* “The number of treatment disrupting events that occurred and were attributable to the presence of the dogs were recorded on fidelity checklists (e.g., barking, whining). Of the 157 completed treatment sessions that included a dog, clinicians identified a treatment disrupting event occurring in 10 of them (6.4%). Six of those events were caused by a single dog who often whined and panted after being separated from his handler. These six events all occurred in the context of the treatment of a single participant, as the dog was removed from further involvement after the case was completed. Removing the 12 sessions involving this dog and his six treatment disrupting events left only four of 145 sessions with such disruptions (2.8%).” [69].

*Explanation:* Reasons for losses or exclusions of the dog(s)/dog handler(s) should be transparently reported (e.g., dog welfare, changes in dog-handler availability), and how these losses/exclusions were initiated (e.g., meeting pre-determined thresholds, dog handler decision). This information informs appropriate dog selection in practice and informs generalisability and replicability. It is recommended to include this information in the CONSORT flow diagram.

### CONSORT 25: A table showing baseline demographic and clinical characteristics for each group

The following two DAI extensions apply to this CONSORT item.

- ➢ **Extension for DAI trials: Baseline data indicating concomitant interactions with dogs (e.g., interactions with pets/other dogs) if relevant. **

*CONSORT example:* “Child and parent participant demographic information was obtained via self-report at baseline (prior to randomization), including the items listed in Table 1. Standard demographic variables were used to describe the sample. Additionally, pet ownership was included as this is a factor that has been thought to influence the impact of AAI.” [134].

*Explanation:* Collecting baseline data on concomitant interactions with dogs is important in understanding confounding effects. Prior experience with dogs may influence participants’ responses to the intervention, impacting outcomes such as engagement, and therefore impact. Identifying these factors informs interpretation of trial findings and generalisability.

- ➢ **Extension for DAI trials: Description of the number and characteristics of the dog(s) and their handler(s) involved.**

**Fig. 2:**
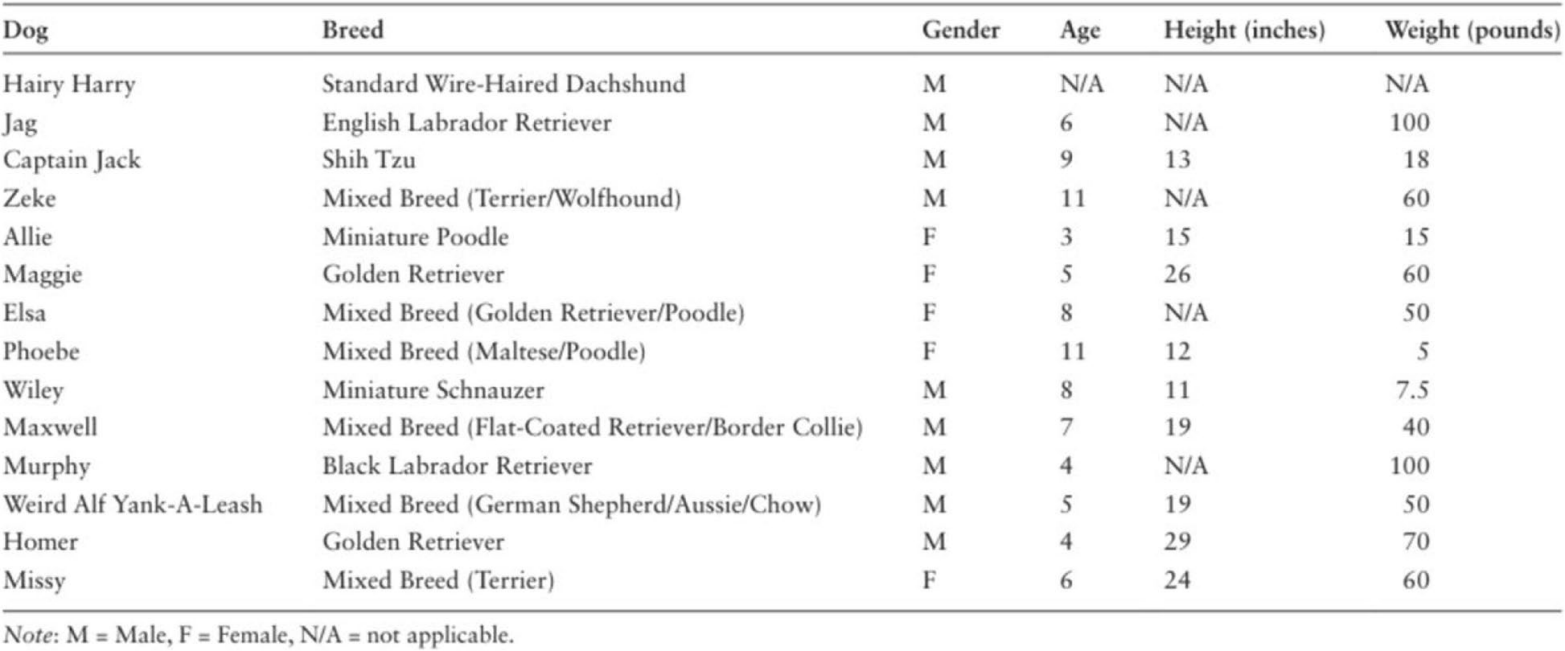
Table illustrating good reporting for CONSORT item 25, DAI extension: Description of the number and characteristics of the dog(s) and their handler(s) involved

*CONSORT example*: “There were 14 DoC dogs [Human-Animal Interaction Dogs On Call program), and 15 human handlers (one dog had two handlers) involved in this study. Of the 15 handlers, 2 were male, 14 were White, 1 was White/Hispanic and the average age of those handlers who reported their age (*n* = 9) was 60.55 years. See Table 3 [see Fig. 2] for demographic information on the dogs.” [133].

*Explanation:* Providing a detailed description of the number and characteristics of the dog(s) and their handlers is important for transparency and replicability. Variations in dog-related factors (e.g., age, size, breed, training level) and handler-related factors (e.g., gender, ethnicity, length of experience) may influence intervention outcomes. This information helps to describe the context of the intervention.

**SPIRIT 34: Provisions, if any, for ancillary and post-trial care, and for compensation to those who suffer harm from trial participation.**

The following two DAI extensions apply to this SPIRIT item.

- ➢ **Extension for DAI trials: Description for post-session and/or post-intervention care in relation to dog wellbeing where applicable.**

A suitable SPIRIT example was not found. Therefore, we have created this example as an illustration of what authors may wish to consider.

*SPIRIT example: “*After completing a session, the dog will be given a walk for at least 20 min and allowed to run free in a secure paddock or indoor area. The handler will provide the dog with the opportunity for interactive play. The walk will only be terminated when the dog voluntarily comes to the handler when gently called and stands quietly to allow the leash to be attached. Dogs will be given a minimum of 30 min between sessions and will be provided with essential resources such as food and water ad lib and a safe haven that they are free to access, and which when occupied by the dog signals their desire to disengage with others. The handler will monitor the dog for signs of stress or fatigue and will withdraw the dog from further sessions if there are concerns about wellbeing. Following the end of the intervention period, the dog’s workload will be reduced for one week.”

*Explanation:* Interventions can be physically demanding (e.g., involving movement, interaction, and prolonged activity) and/or emotionally taxing for dogs (e.g., if they encounter unfamiliar people or high energy environments). Post-session and post-intervention care ensures they have time to rest, hydrate, and recover. Without adequate post-session or post-intervention care, dogs may experience cumulative stress or fatigue, which can lead to burnout and reduce their ability to continue participating in interventions. After sessions, dogs should be checked for signs of injury, fatigue, or illness to address any issues promptly.

- ➢ **Extension for DAI trials: Provisions for supporting the end of interactions between the dog and participant (post-session and/or post-intervention).**

A suitable SPIRIT example was not found. Therefore, we have created this example as an illustration of what authors may wish to consider.

SPIRIT example: “For the last 5 min of each session, time will be allocated for a structured closure to the interaction between participant and dog. This will include a brief activity of petting/brushing the dog, and reflecting on the session, supporting emotional regulation of the participant and allowing the dog to have a predictable conclusion to each interaction. At the end of the full intervention period, a final session will include a planned farewell activity in which the participant is given the opportunity to write a note or create a drawing for the dog and is given a photo of the dog to keep. During this final session the participant will be offered space to share any feelings or questions that might arise in relation to the ending of the relationship, recognising that some participants may experience a sense of loss”.

*Explanation:* Participants often develop strong emotional bonds with therapy dogs [135]. An abrupt or poorly managed end to the interaction can lead to sadness, confusion, or emotional distress, particularly for vulnerable populations like children, older adults, or those with mental health conditions. Considering and implementing a supported process for saying goodbye can help participants process the end of the interaction in a healthy way, potentially reducing feelings of loss or abandonment [127]. Guided conversations or activities at the end of a session can help participants reflect on their experiences, reinforcing positive outcomes and learnings [128, 129].

Dogs may also experience stress or confusion during transitions if goodbyes are abrupt or chaotic. Clear protocols ensure these moments are calm and positive for the dog. Properly managing endings helps avoid situations where participants cling to the dog, potentially overburdening or distressing the dog.

## Conclusion

DAI trials present unique methodological and reporting challenges due to the involvement of animals, variability in intervention delivery, and complexities related to participant-dog interactions. These trials require careful consideration in both design and reporting to ensure transparency, replicability, and scientific rigour. However, reviews of DAI trials have revealed substantial shortcomings in reporting quality, limiting the interpretability and generalisability of the findings [31]. The SPIRIT 2025 and CONSORT 2025 extensions for DAI trials presented here offer tailored guidance to support researchers in improving the reporting of both trial protocols and completed trial reports. This E&E paper provides a detailed rationale and illustrative examples to aid authors in the consistent implementation of these extensions.

## Data Availability

No datasets were generated or analysed during the current study.

## References

[1] Chan AW, Tetzlaff JM, Altman DG, Laupacis A, Gøtzsche PC, Krleža-Jerić K, et al. SPIRIT 2013 statement: defining standard protocol items for clinical trials. Ann Intern Med. 2013;158(3):200–7. 23295957 10.7326/0003-4819-158-3-201302050-00583 PMC5114123

[2] Chan AW, Tetzlaff JM, Gøtzsche PC, Altman DG, Mann H, Berlin JA, et al. SPIRIT 2013 explanation and elaboration: guidance for protocols of clinical trials. BMJ. 2013;346:e7586. 23303884 10.1136/bmj.e7586 PMC3541470

[3] Moher D, Hopewell S, Schulz KF, Montori V, Gøtzsche PC, Devereaux PJ, et al. CONSORT 2010 explanation and elaboration: updated guidelines for reporting parallel group randomised trials. Int J Surg. 2012;10(1):28–55. 22036893 10.1016/j.ijsu.2011.10.001

[4] Schulz KF, Altman DG, Moher D. CONSORT 2010 statement: updated guidelines for reporting parallel group randomised trials. Trials. 2010;11(1):1–8. 21350618 10.4103/0976-500X.72352 PMC3043330

[5] Hopewell S, Chan AW, Collins GS, Hróbjartsson A, Moher D, Schulz KF, et al. CONSORT 2025 explanation and elaboration: updated guideline for reporting randomised trials. BMJ. 2025;389:e081124. 40228832 10.1136/bmj-2024-081124 PMC11995452

[6] Chan, A.W., Boutron, I., Hopewell, S., Moher, D., Schulz, K.F., Collins, G.S., Tunn, R., Aggarwal, R., Berkwits, M., Berlin, J.A. and Bhandari, N., 2025. SPIRIT 2025 statement: updated guideline for protocols of randomised trials. The Lancet, 405(10491), pp.e19-e27.10.1016/S0140-6736(25)00770-6 10.1371/journal.pmed.1004589 PMC12037212 40294521

[7] Tan ZW, Tan AC, Li T, Harris I, Naylor JM, Siebelt M, et al. Has the reporting quality of published randomised controlled trial protocols improved since the SPIRIT statement? A methodological study. BMJ Open. 2020;10(8):e038283. 32847919 10.1136/bmjopen-2020-038283 PMC7451949

[8] Moher D, Jones A, Lepage L, Group C. Use of the CONSORT statement and quality of reports of randomized trials: a comparative before-and-after evaluation. JAMA. 2001;285(15):1992–5. 11308436 10.1001/jama.285.15.1992

[9] Plint AC, Moher D, Morrison A, Schulz K, Altman DG, Hill C, et al. Does the CONSORT checklist improve the quality of reports of randomised controlled trials? A systematic review. Med J Aust. 2006;185(5):263–7. 16948622 10.5694/j.1326-5377.2006.tb00557.x

[10] Turner L, Shamseer L, Altman DG, Weeks L, Peters J, Kober T, et al. Consolidated standards of reporting trials (CONSORT) and the completeness of reporting of randomised controlled trials (RCTs) published in medical journals. Cochrane database of systematic reviews. 2012(11). 10.1002/14651858.MR000030.pub2 PMC7386818 23152285

[11] Devereaux P, Manns BJ, Ghali WA, Quan H, Guyatt GH. The reporting of methodological factors in randomized controlled trials and the association with a journal policy to promote adherence to the consolidated standards of reporting trials (CONSORT) checklist. Control Clin Trials. 2002;23(4):380–8. 12161081 10.1016/s0197-2456(02)00214-3

[12] Shamseer L, Hopewell S, Altman DG, Moher D, Schulz KF. Update on the endorsement of CONSORT by high impact factor journals: a survey of journal “Instructions to Authors” in 2014. Trials. 2016;17:1–8. 27343072 10.1186/s13063-016-1408-z PMC4921029

[13] Wang P, Wolfram D, Gilbert E. Endorsements of five reporting guidelines for biomedical research by journals of prominent publishers. PLoS One. 2024;19(2):e0299806. 38421981 10.1371/journal.pone.0299806 PMC10903802

[14] Hopewell S, Altman DG, Moher D, Schulz KF. Endorsement of the CONSORT statement by high impact factor medical journals: a survey of journal editors and journal’instructions to authors’. Trials. 2008;9:1–7. 18423021 10.1186/1745-6215-9-20 PMC2359733

[15] Campbell MK, Piaggio G, Elbourne DR, Altman DG, Group C. Consort 2010 statement: extension to cluster randomised trials. BMJ. 2012;345:e5661. 22951546 10.1136/bmj.e5661

[16] Piaggio G, Elbourne DR, Pocock SJ, Evans SJ, Altman DG, Group C. Reporting of noninferiority and equivalence randomized trials: extension of the CONSORT 2010 statement. JAMA. 2012;308(24):2594–604. 23268518 10.1001/jama.2012.87802

[17] Dwan K, Li T, Altman DG, Elbourne D. CONSORT 2010 statement: extension to randomised crossover trials. BMJ. 2019;366:l4378. 31366597 10.1136/bmj.l4378 PMC6667942

[18] Hemming K, Taljaard M, McKenzie JE, Hooper R, Copas A, Thompson JA, et al. Reporting of stepped wedge cluster randomised trials: extension of the CONSORT 2010 statement with explanation and elaboration. BMJ. 2018;363:k1614. 30413417 10.1136/bmj.k1614 PMC6225589

[19] Boutron I, Moher D, Altman DG, Schulz KF, Ravaud P, Group. Methods and processes of the CONSORT Group: example of an extension for trials assessing nonpharmacologic treatments. Ann Intern Med. 2008;148(4):W-60-W−6. 18283201 10.7326/0003-4819-148-4-200802190-00008-w1

[20] Grant S, on behalf of the C-SPIG. The CONSORT-SPI 2018 extension: a new guideline for reporting social and psychological intervention trials. Addiction. 2019;114(1):4–8. 30091280 10.1111/add.14411

[21] Grant SP, Mayo-Wilson E, Melendez-Torres G, Montgomery P. Reporting quality of social and psychological intervention trials: a systematic review of reporting guidelines and trial publications. PLoS One. 2013;8(5):e65442. 23734256 10.1371/journal.pone.0065442 PMC3666983

[22] Craig P, Dieppe P, Macintyre S, Michie S, Nazareth I, Petticrew M. Developing and evaluating complex interventions: the new Medical Research Council guidance. Bmj. 2008;337. 10.1136/bmj.a1655 PMC2769032 18824488

[23] Michie, S., Fixsen, D., Grimshaw, J.M. and Eccles, M.P., 2009. Specifying and reporting complex behaviour change interventions: the need for a scientific method. Implementation science, 4(1), p.40.10.1186/1748-5908-4-40 10.1186/1748-5908-4-40 PMC2717906 19607700

[24] Mayo-Wilson E. Reporting implementation in randomized trials: proposed additions to the consolidated standards of reporting trials statement. Am J Public Health. 2007;97(4):630–3. 17329641 10.2105/AJPH.2006.094169 PMC1829360

[25] Glasziou P, Meats E, Heneghan C, Shepperd S. What is missing from descriptions of treatment in trials and reviews? BMJ. 2008;336(7659):1472–4. 18583680 10.1136/bmj.39590.732037.47 PMC2440840

[26] Glasziou P, Altman DG, Bossuyt P, Boutron I, Clarke M, Julious S, et al. Reducing waste from incomplete or unusable reports of biomedical research. Lancet. 2014;383(9913):267–76. 24411647 10.1016/S0140-6736(13)62228-X

[27] Moher D, Glasziou P, Chalmers I, Nasser M, Bossuyt PM, Korevaar DA, et al. Increasing value and reducing waste in biomedical research: who’s listening? Lancet. 2016;387(10027):1573–86. 26423180 10.1016/S0140-6736(15)00307-4

[28] Driessen E, Hollon SD, Bockting CL, Cuijpers P, Turner EH. Does publication bias inflate the apparent efficacy of psychological treatment for major depressive disorder? A systematic review and meta-analysis of US National Institutes of Health-funded trials. PLoS One. 2015;10(9):e0137864. 26422604 10.1371/journal.pone.0137864 PMC4589340

[29] Nimer J, Lundahl B. Animal-assisted therapy: a meta-analysis. Anthrozoos. 2007;20(3):225–38.

[30] Ratschen E, Sheldon TA. Elephant in the room: animal assisted interventions. BMJ. 2019;367:l6260. 31848134 10.1136/bmj.l6260

[31] Shoesmith, E., Hall, S., Sowden, A., Stevens, H., Pervin, J., Riga, J., McMillan, D., Mills, D., Clarke, C., Wu, Q. and Gibsone, S., 2025. Dog-assisted interventions for children and adults with mental health or neurodevelopmental conditions: systematic review. The British Journal of Psychiatry, pp.1-14. 10.1192/bjp.2025.8 10.1192/bjp.2025.8 PMC7617605 40223561

[32] Gómez-Calcerrada I, Lavín-Pérez AM, Villafaina S, Rueda-Rubio JC, Rivera-Martín B, González-García I, et al. Effects of dog-assisted therapy on the physical function and communication skills of adults with autism: a study protocol for a controlled study. Appl Sci. 2021;11(22):10650.

[33] Vidal R, Vidal L, Lugo J, Ristol F, Domenec E, Casas T, et al. Dog-assisted therapy vs relaxation for children and adolescents with fetal alcohol spectrum disorder: a randomized controlled study. J Autism Dev Disord. 2023. 10.1007/s10803-023-06023-5. 37340213 10.1007/s10803-023-06023-5

[34] Lundqvist M, Carlsson P, Sjodahl R, Theodorsson E, Levin LÅ. Patient benefit of dog-assisted interventions in health care: a systematic review. BMC Complement Altern Med. 2017;17(1):358. 28693538 10.1186/s12906-017-1844-7 PMC5504801

[35] Badin L, Alibran E, Pothier K, Bailly N. Effects of equine-assisted interventions on older adults’ health: a systematic review. Int J Nurs Sci. 2022;9(4):542–52. 36285074 10.1016/j.ijnss.2022.09.008 PMC9587396

[36] Shoesmith E, Surr C, Ratschen E. Animal-assisted and robotic animal-assisted interventions within dementia care: a systematic review. Dementia. 2023;22(3):664–93. 36765455 10.1177/14713012231155985 PMC10014823

[37] Nieforth LO, Schwichtenberg AJ, O’Haire ME. Animal-assisted interventions for autism spectrum disorder: a systematic review of the literature from 2016 to 2020. Rev J Autism Dev Disord. 2023. 10.1007/s40489-021-00291-6. 37313251 10.1007/s40489-021-00291-6 PMC10259834

[38] Suba-Bokodi É, Nagy I, Molnár M. Unconventional animal species participation in animal-assisted interventions and methods for measuring their experienced stress. Animals. 2024;14(20):2935. 39457864 10.3390/ani14202935 PMC11503701

[39] Kruger, K.A. and Serpell, J.A., 2010. Animal-assisted interventions in mental health: Definitions and theoretical foundations. In Handbook on animal-assisted therapy (pp. 33-48). Academic Press.10.1016/B978-0-12-381453-1.10003-0

[40] Fine, A.H. (ed.) (2019) Handbook on Animal-Assisted Therapy: Foundations and Guidelines for Animal-Assisted Interventions. 5th edn. London: Academic Press. ISBN: 9780128153956.

[41] Brelsford VL, Meints K, Gee NR, Pfeffer K. Animal-assisted interventions in the classroom—a systematic review. Int J Environ Res Public Health. 2017;14(7):669. 28640200 10.3390/ijerph14070669 PMC5551107

[42] Binder AJ, Parish-Plass N, Kirby M, Winkle M, Skwerer DP, Ackerman L, et al. Recommendations for uniform terminology in animal-assisted services (AAS). Human-Animal Interactions. 2024;12(1).

[43] Hill J, Ziviani J, Cawdell-Smith J, Driscoll C. Canine assisted occupational therapy: Protocol of a pilot randomised control trial for children on the autism spectrum. Open J Pediatr. 2019;9(03):199. 10.1007/s10803-020-04483-7 32266682

[44] Briones MA, Pardo-Garcia I, Escribano-Sotos F. Effectiveness of a dog-assisted therapy program to enhance quality of life in institutionalized dementia patients. Clin Nurs Res. 2019. 10.1177/1054773819867250. 31387390 10.1177/1054773819867250

[45] Arnskotter W, Martin S, Walitza S, Hediger K. Effects of including a dog on treatment motivation and the therapeutic alliance in child and adolescent psychotherapy: study protocol for a randomized controlled trial. Trials. 2024;25(1):26. 38183121 10.1186/s13063-023-07854-4 PMC10768352

[46] IAHAIO (2018) IAHAIO White Paper: Definitions for Animal Assisted Intervention and Guidelines for Wellness of Animals Involved. Updated April 2018. IAHAIO. Available at: https://iahaio.org/wp/wp-content/uploads/2018/04/iahaio_wp_updated-2018-final.pdf . Accessed 1 Sep 2025

[47] Seivert NP, Cano A, Casey RJ, May DK, Johnson A. Animal assisted therapy for incarcerated youth: a randomized controlled trial. Appl Dev Sci. 2018;22(2):139–53. 30906186 10.1080/10888691.2016.1234935 PMC6430139

[48] Walker H, Brisman R, Miller MC, Cowfer B, Akard T, Gilmer MJ. The effects of animal-assisted interactions on quality of life in children with life-threatening conditions and their parents. Int J Palliat Nurs. 2021;27(10):524–30. 34919414 10.12968/ijpn.2021.27.10.524

[49] Wijker C, Leontjevas R, Spek A, Enders-Slegers M-J. Effects of dog assisted therapy for adults with autism spectrum disorder: an exploratory randomized controlled trial. J Autism Dev Disord. 2020;50(6):2153–63. 30900194 10.1007/s10803-019-03971-9 PMC7261269

[50] O’Haire ME. Animal-assisted intervention for autism spectrum disorder: a systematic literature review. J Autism Dev Disord. 2013;43(7):1606–22. 23124442 10.1007/s10803-012-1707-5

[51] Gabriels RL, Pan Z, Dechant B, Agnew JA, Brim N, Mesibov G. Randomized controlled trial of therapeutic horseback riding in children and adolescents with autism spectrum disorder. J Am Acad Child Adolesc Psychiatry. 2015;54(7):541–9. 26088658 10.1016/j.jaac.2015.04.007 PMC4475278

[52] Vreeburg SA, Zitman FG, van Pelt J, DeRijk RH, Verhagen JC, van Dyck R, et al. Salivary cortisol levels in persons with and without different anxiety disorders. Psychosom Med. 2010;72(4):340–7. 20190128 10.1097/PSY.0b013e3181d2f0c8

[53] Beetz A, Uvnas-Moberg K, Julius H, Kotrschal K. Psychosocial and psychophysiological effects of human-animal interactions: The possible role of oxytocin. Front Psychol. 2012;3. 10.3389/fpsyg.2012.00234 PMC3408111 22866043

[54] Cooper K, Smith LG, Russell A. Social identity, self-esteem, and mental health in autism. Eur J Soc Psychol. 2017;47(7):844–54.

[55] IAHAIO (2014) IAHAIO White Paper: The IAHAIO Definitions for Animal Assisted Intervention and Guidelines for Wellness of Animals Involved. Final Report of the IAHAIO Task Force, July 2014. Available at: https://www.esadoggy.com/wp-content/uploads/2018/07/8000IAHAIO-WHITE-PAPER-TASK-FORCE-FINAL-REPORT-070714.pdf. Accessed 1 Sep 2025

[56] Banks MR. The effects of animal-assisted therapy on loneliness in an elderly population in long-term care facilities. Dissertation Abstracts International: Section B: The Sciences and Engineering. 1998;59(3-B):1043. 10.1093/gerona/57.7.m428 12084804

[57] Levinson BM. Human/companion animal therapy. J Contemp Psychother. 1984;14(2):131–44.

[58] Brickel CM. Initiation and maintenance of the human-animal bond: familial roles from a learning perspective. Marriage Fam Rev. 1985;8(3–4):31–48.

[59] Odendaal JS. Animal-assisted therapy - magic or medicine? J Psychosom Res. 2000;49(4):275–80. 11119784 10.1016/s0022-3999(00)00183-5

[60] Tabares C, Vicente F, Sánchez S, Aparicio A, Alejo S, Cubero J. Quantification of hormonal changes by effects of hippotherapy in the autistic population. Neurochem J. 2012;6(4):311–6.

[61] .Wijker, C., Spek, A.A., Leontjevas, R., Verheggen, T. and Enders-Slegers, M.J., 2017. The effectiveness of animal assisted therapy in adults with autism spectrum disorder: Study protocol for a randomized controlled trial. Autism Open Access (7:5) , page 1-8. 10.4172/2165-7890.1000221

[62] Odendaal JSJ, Meintjes RA. Neurophysiological correlates of affiliative behaviour between humans and dogs. Vet J. 2003;165(3):296–301. 12672376 10.1016/s1090-0233(02)00237-x

[63] Wijker C, Kupper N, Leontjevas R, Spek A, Enders-Slegers MJ. The effects of animal assisted therapy on autonomic and endocrine activity in adults with autism spectrum disorder: a randomized controlled trial. Gen Hosp Psychiatry. 2021;72:36–44. 34237553 10.1016/j.genhosppsych.2021.05.003

[64] Teo JT, Johnstone SJ, Romer SS, Thomas SJ. Psychophysiological mechanisms underlying the potential health benefits of human-dog interactions: a systematic literature review. Int J Psychophysiol. 2022;180:27–48. 35901904 10.1016/j.ijpsycho.2022.07.007

[65] Lewis CC, Boyd MR, Walsh-Bailey C, Lyon AR, Beidas R, Mittman B, et al. A systematic review of empirical studies examining mechanisms of implementation in health. Implement Sci. 2020;15(1):21. 32299461 10.1186/s13012-020-00983-3 PMC7164241

[66] Wagner C, Gaab J, Hediger K. The importance of the treatment rationale for pain in animal-assisted interventions: a randomized controlled trial in healthy participants. J Pain. 2023;24(6):1080–93. 36641027 10.1016/j.jpain.2023.01.004

[67] Allen B, Shenk CE, Dreschel NE, Wang M, Bucher AM, Desir MP, et al. Integrating animal-assisted therapy into TF-CBT for abused youth with PTSD: a randomized controlled feasibility trial. Child Maltreat. 2022;27(3):466–77. 33499659 10.1177/1077559520988790 PMC9215110

[68] Schuck SEB, Johnson HL, Abdullah MM, Stehli A, Fine AH, Lakes KD. The role of animal assisted intervention on improving self-esteem in children with attention deficit/hyperactivity disorder. Front Pediatr. 2018. 10.3389/fped.2018.00300. 30450352 10.3389/fped.2018.00300 PMC6224337

[69] Villalta-Gil V, Roca M, Gonzalez N, Domènec E, Cuca, Escanilla A, et al. Dog-assisted therapy in the treatment of chronic schizophrenia Inpatients. Anthrozoös. 2009;22(2):149–59.

[70] Wolynczyk-Gmaj D, Ziolkowska A, Rogala P, Scigala D, Bryla L, Gmaj B, et al. Can dog-assisted intervention decrease anxiety level and autonomic agitation in patients with anxiety disorders? J Clin Med. 2021;10(21):5171. 34768691 10.3390/jcm10215171 PMC8584515

[71] Lobato Rincón LL, Rivera Martín B, Medina Sánchez MÁ, Villafaina S, Merellano-Navarro E, Collado-Mateo D. Effects of dog-assisted education on physical and communicative skills in children with severe and multiple disabilities: a pilot study. Animals. 2021;11(6):1741. 34200895 10.3390/ani11061741 PMC8230480

[72] Lavin-Perez AM, Martin-Sanchez C, Martinez-Nunez B, Lobato-Rincon LL, Villafaina S, Gonzalez-Garcia I, et al. Effects of dog-assisted therapy in adolescents with eating disorders: a study protocol for a pilot controlled trial. Animals. 2021;11(10):2784. 34679805 10.3390/ani11102784 PMC8532616

[73] Gomez-Calcerrada I, Lavin-Perez AM, Villafaina S, Rueda-Rubio JC, Rivera-Martin B, Gonzalez-Garcia I, et al. Effects of dog-assisted therapy on the physical function and communication skills of adults with autism: a study protocol for a controlled study. Appl Sci. 2021. 10.3390/app112210650.

[74] Binfet JT, Tardif-Williams C, Draper ZA, Green FLL, Singal A, Rousseau CX, et al. Virtual canine comfort: a randomized controlled trial of the effects of a canine-assisted intervention supporting undergraduate wellbeing. Anthrozoos. 2022;35(6):809–32.

[75] Palestrini C, Calcaterra V, Cannas S, Talamonti Z, Papotti F, Buttram D, et al. Stress level evaluation in a dog during animal-assisted therapy in pediatric surgery. J Vet Behav. 2017;17:44–9.

[76] Glenk LM. Current perspectives on therapy dog welfare in animal-assisted interventions. Animals. 2017;7(2):7. 28157145 10.3390/ani7020007 PMC5332928

[77] Heimlich K. Animal-assisted therapy and the severely disabled child: a quantitative study. J Rehabil. 2001;67(4).

[78] Clark SD, Martin F, McGowan RTS, Smidt JM, Anderson R, Wang L, et al. Physiological state of therapy dogs during animal-assisted activities in an outpatient setting. Animals. 2020;10(5):819. 32397366 10.3390/ani10050819 PMC7277909

[79] Miller SL, Serpell JA, Dalton KR, Waite KB, Morris DO, Redding LE, et al. The importance of evaluating positive welfare characteristics and temperament in working therapy dogs. Front Vet Sci. 2022;9:844252. 35445102 10.3389/fvets.2022.844252 PMC9014261

[80] Stevenson K, Jarred S, Hinchcliffe V, Roberts K. Can a dog be used as a motivator to develop social interaction and engagement with teachers for students with autism? Support Learn. 2015;30(4):341–63.

[81] Serpell, J.A., McCune, S., Gee, N.R. and Griffin, J.A. (2017) ‘From the animal’s perspective: Welfare implications of human–animal interaction research and practice’, in Fine, A.H. (ed.) Handbook on Animal-Assisted Therapy: Foundations and Guidelines for Animal-Assisted Interventions. 4th edn. San Diego, CA: Academic Press, pp. 185–204. Available at: https://psycnet.apa.org/record/2015-40807-013. Accessed 1 Sep 2025.

[82] Massey NG, Wheeler J. Acquisition and generalization of activity schedules and their effects on task engagement in a young child with autism in an inclusive pre-school classroom. Educ Train Ment Retard Dev Disabil. 2000;35:326–35.

[83] Mongillo P, Pitteri E, Adamelli S, Bonichini S, Farina L, Marinelli L. Validation of a selection protocol of dogs involved in animal assisted intervention. J Vet Behav Clin Appl Res. 2014;10.

[84] Bono AV, Benvenuti C, Buzzi M, Ciatti R, Chiarelli V, Chiambretto P, et al. Effects of animal assisted therapy (AAT) carried out with dogs on the evolution of mild cognitive impairment. G Gerontol. 2015;63(1):32–6.

[85] Winkle M, Johnson A, Mills D. Dog welfare, well-being and behavior: considerations for selection, evaluation and suitability for animal-assisted therapy. Animals. 2020. 10.3390/ani10112188. 33238376 10.3390/ani10112188 PMC7700550

[86] Chen CR, Hung CF, Lee YW, Tseng WT, Chen ML, Chen TT. Functional outcomes in a randomized controlled trial of animal-assisted therapy on middle-aged and older adults with schizophrenia. Int J Environ Res Public Health. 2022;19(10):6270. 35627807 10.3390/ijerph19106270 PMC9141906

[87] Mongillo P, Pitteri E, Adamelli S, Bonichini S, Farina L, Marinelli L. Validation of a selection protocol of dogs involved in animal-assisted intervention. J Vet Behav. 2015;10(2):103–10.

[88] Wolynczyk-Gmaj D, Ziolkowska A, Rogala P, Scigala D, Bryla L, Gmaj B, et al. Can Dog-assisted intervention decrease anxiety level and autonomic agitation in patients with anxiety disorders? J Clin Med. 2021;10(21). 10.3390/jcm10215171 PMC8584515 34768691

[89] Guymer EC, Mellor D, Luk ES, Pearse V. The development of a screening questionnaire for childhood cruelty to animals. J Child Psychol Psychiatry. 2001;42(8):1057–63. 11806688 10.1111/1469-7610.00805

[90] Baek SM, Lee Y, Sohng KY. The psychological and behavioural effects of an animal-assisted therapy programme in Korean older adults with dementia. Psychogeriatrics. 2020;20(5):645–53. 32291838 10.1111/psyg.12554 PMC7586947

[91] Chen TT, Hsieh TL, Chen ML, Tseng WT, Hung CF, Chen CR. Animal-assisted therapy in middle-aged and older patients with schizophrenia: a randomized controlled trial. Front Psychiatry. 2021;12:713623. 34456769 10.3389/fpsyt.2021.713623 PMC8386276

[92] Quintavalla F, Cao S, Spinelli D, Caffarra P, Rossi FM, Basini G, et al. Effects of dog-assisted therapies on cognitive mnemonic capabilities in people affected by Alzheimer’s disease. Animals. 2021;11(5):1366. 34064930 10.3390/ani11051366 PMC8151255

[93] Menna LF, Santaniello A, Amato A, Ceparano G, Di Maggio A, Sansone M, et al. Changes of oxytocin and serotonin values in dialysis patients after animal assisted activities (AAAs) with a dog—a preliminary study. Animals. 2019;9(8):526. 31382576 10.3390/ani9080526 PMC6721151

[94] Hill J, Ziviani J, Driscoll C, Teoh AL, Chua JM, Cawdell-Smith J. Canine assisted occupational therapy for children on the autism spectrum: a pilot randomised control trial. J Autism Dev Disord. 2020;50(11):4106–20. 32266682 10.1007/s10803-020-04483-7

[95] Henderson L, Grové C, Lee F, Trainer L, Schena H, Prentice M. An evaluation of a dog-assisted reading program to support student wellbeing in primary school. Child Youth Serv Rev. 2020;118:N.PAG-N.PAG.

[96] Calvo, P., Fortuny, J.R., Guzmán, S., Macías, C., Bowen, J., García, M.L., Orejas, O., Molins, F., Tvarijonaviciute, A., Cerón, J.J. and Bulbena, A., 2016. Animal assisted therapy (AAT) program as a useful adjunct to conventional psychosocial rehabilitation for patients with schizophrenia: results of a small-scale randomized controlled trial. Frontiers in psychology, 7, p.631.10.3389/fpsyg.2016.00631 10.3389/fpsyg.2016.00631 PMC4858645 27199859

[97] Olsen C, Pedersen I, Bergland A, Enders-Slegers M-J, Patil G, Ihlebaek C. Effect of animal-assisted interventions on depression, agitation and quality of life in nursing home residents suffering from cognitive impairment or dementia: a cluster randomized controlled trial. Int J Geriatr Psychiatry. 2016;31(12):1312–21. 26807956 10.1002/gps.4436

[98] Shih CA, Yang MH. Effect of animal-assisted therapy (AAT) on social interaction and quality of life in patients with schizophrenia during the COVID-19 pandemic: an experimental study. Asian Nurs Res. 2023;17(1):37–43. 10.1016/j.anr.2023.01.002 PMC9837379 36646276

[99] Hill J, Ziviani J, Driscoll C, Cawdell-Smith J. Canine-assisted occupational therapy for children on the autism spectrum: challenges in practice. Br J Occup Ther. 2020;83(4):215–9 (Special Issue: Optimizing children&apos;s participation for health and wellbeing II: Barriers and facilitators.).

[100] Scorzato I, Zaninotto L, Romano M, Menardi C, Cavedon L, Pegoraro A, et al. Effects of dog-assisted therapy on communication and basic social skills of adults with intellectual disabilities: a pilot study. Intellect Dev Disabil. 2017;55(3):125–39. 28608768 10.1352/1934-9556-55.3.125

[101] Curran BJ, Jenkins MA. The Global and Cross-Cultural Reach of Trauma-Informed Animal-Assisted Interventions. New Directions In The Human-Animal Bond. 2019:423.

[102] Dearden J, Emerson A, Lewis T, Papp R. Transforming engagement: a case study of building intrinsic motivation in a child with autism. Br J Spec Educ. 2016;44:8–25.

[103] Sams MJ, Fortney EV, Willenbring S. Occupational therapy incorporating animals for children with autism: a pilot investigation. Am J Occup Ther. 2006;60(3):268–74. 16776394 10.5014/ajot.60.3.268

[104] Case-Smith, J. (2010) ‘An overview of occupational therapy for children’, in Case-Smith, J. and O’Brien, J. (eds.) Occupational Therapy for Children. 6th edn. Amsterdam: Elsevier, pp. 3–25

[105] Ng Z, Albright J, Fine A, Peralta J. Our Ethical and Moral Responsibility. 2019. Academic Press, London p. 175–98.10.1016/B978-0-12-801292-5.00026-2

[106] Braun C, Stangler T, Narveson J, Pettingell S. Animal-assisted therapy as a pain relief intervention for children. Complement Ther Clin Pract. 2009;15(2):105–9. 19341990 10.1016/j.ctcp.2009.02.008

[107] Marcus DA, Bernstein CD, Constantin JM, Kunkel FA, Breuer P, Hanlon RB. Impact of animal-assisted therapy for outpatients with fibromyalgia. Pain Med. 2013;14(1):43–51. 23170993 10.1111/j.1526-4637.2012.01522.x PMC3666031

[108] Barker SB, Dawson KS. The effects of animal-assisted therapy on anxiety ratings of hospitalized psychiatric patients. Psychiatr Serv. 1998;49(6):797–801. 9634160 10.1176/ps.49.6.797

[109] Sobo EJ, Eng B, Kassity-Krich N. Canine visitation (pet) therapy: pilot data on decreases in child pain perception. J Holist Nurs. 2006;24(1):51–7. 16449747 10.1177/0898010105280112

[110] Kertes DA, Liu J, Hall NJ, Hadad NA, Wynne CDL, Bhatt SS. Effect of pet dogs on children’s perceived stress and cortisol stress response. Soc Dev. 2017;26(2):382–401. 28439150 10.1111/sode.12203 PMC5400290

[111] Kline JA, Fisher MA, Pettit KL, Linville CT, Beck AM. Controlled clinical trial of canine therapy versus usual care to reduce patient anxiety in the emergency department. PLoS One. 2019;14(1):e0209232. 30625184 10.1371/journal.pone.0209232 PMC6326463

[112] Ng Z, Svensson L, Souza M, Albright J. Describing adverse events in an animal-assisted intervention organization: Recommendations for prevention and management. Human-Animal Interactions. 2024 Apr 4(2024). 10.1079/hai.2024.0011

[113] Olsen C, Pedersen I, Bergland A, Enders-Slegers MJ, Ihlebak C. Effect of animal-assisted activity on balance and quality of life in home-dwelling persons with dementia. Geriatric nursing (New York, NY). 2016;37(4):284–91. 10.1016/j.gerinurse.2016.04.002 27155968

[114] Calvo P, Fortuny JR, Guzmán S, Macías C, Bowen J, García ML, et al. Animal assisted therapy (AAT) program as a useful adjunct to conventional psychosocial rehabilitation for patients with schizophrenia: results of a small-scale randomized controlled trial. Front Psychol. 2016;7:631. 27199859 10.3389/fpsyg.2016.00631 PMC4858645

[115] Verkerk G, Wolf M, Louwers A, Meester-Delver A, Nollet F. The reproducibility and validity of the Canadian Occupational Performance Measure in parents of children with disabilities. Clin Rehabil. 2006;20:980–8. 17065541 10.1177/0269215506070703

[116] Krause-Parello CA, Friedmann E. The effects of an animal-assisted intervention on salivary alpha-amylase, salivary immunoglobulin A, and heart rate during forensic interviews in child sexual abuse cases. Anthrozoos. 2014;27(4):581–90.

[117] MacLean EL, Gesquiere LR, Gee N, Levy K, Martin WL, Carter CS. Validation of salivary oxytocin and vasopressin as biomarkers in domestic dogs. J Neurosci Methods. 2018;293:67–76. 28865986 10.1016/j.jneumeth.2017.08.033

[118] Ng Z, Albright J, Fine AH, Peralta J. Chapter 26 - Our Ethical and Moral Responsibility: Ensuring the Welfare of Therapy Animals. In: Fine AH, editor. Handbook on Animal-Assisted Therapy. 4th ed. San Diego: Academic Press; 2015. p. 357–76.

[119] Salimetrics. Collection Method: SalivaBio Children’s Swab (SCS). 2017 [Available from: https://www.salimetrics.com/assets/documents/children-swab-saliva-collection-instructions.pdf.

[120] MacLean EL, Gesquiere LR, Gee NR, Levy K, Martin WL, Carter CS. Effects of affiliative human-animal interaction on dog salivary and plasma oxytocin and vasopressin. Front Psychol. 2017;8:1606. 28979224 10.3389/fpsyg.2017.01606 PMC5611686

[121] Torres SM, Furrow E, Souza CP, Granick JL, De Jong EP, Griffin TJ, et al. Salivary proteomics of healthy dogs: An in depth catalog. PLoS ONE. 2018;13(1):e0191307. 29329347 10.1371/journal.pone.0191307 PMC5766244

[122] Hill J, Driscoll C, Cawdell-Smith J, Anderson S, Ziviani J. Investigating dog welfare when interacting with autistic children within canine-assisted occupational therapy sessions: a single case study. Animals. 2023;13(12):1965. 37370475 10.3390/ani13121965 PMC10294977

[123] Meints K, Brelsford VL, Dimolareva M, Marechal L, Pennington K, Rowan E, et al. Can dogs reduce stress levels in school children? Effects of dog-assisted interventions on salivary cortisol in children with and without special educational needs using randomized controlled trials. PLoS One. 2022;17(6 June):e0269333. 35704561 10.1371/journal.pone.0269333 PMC9200172

[124] Stefanini MC, Martino A, Allori P, Galeotti F, Tani F. The use of animal-assisted therapy in adolescents with acute mental disorders: a randomized controlled study. Complement Ther Clin Pract. 2015;21(1):42–6. 25701449 10.1016/j.ctcp.2015.01.001

[125] Stefanini MC, Martino A, Bacci B, Tani F. The effect of animal-assisted therapy on emotional and behavioral symptoms in children and adolescents hospitalized for acute mental disorders. Eur J Integr Med. 2016;8(2):81–8.

[126] Lefebvre SL, Golab GC, Castrodale L, Aureden K, Bialachowski A, Gumley N, et al. Guidelines for animal-assisted interventions in health care facilities. Am J Infect Control. 2008;36(2):78–85. 18313508 10.1016/j.ajic.2007.09.005

[127] Brelsford VL, Dimolareva M, Gee NR, Meints K. Best practice standards in animal-assisted interventions: how the LEAD risk assessment tool can help. Animals. 2020;10(6):1–11. 10.3390/ani10060974 PMC7341237 32503309

[128] Brelsford VL, Dimolareva M, Rowan E, Gee NR, Meints K. Can dog-assisted and relaxation interventions boost spatial ability in children with and without special educational needs? A longitudinal, randomized controlled trial. Front Pediatr. 2022. 10.3389/fped.2022.886324. 35979404 10.3389/fped.2022.886324 PMC9376734

[129] Gee NR, Townsend L, Friedmann E, Barker SB, Mueller MK. A pilot randomized controlled trial to examine the impact of a therapy dog intervention on loneliness in hospitalized older adults. Innov Aging. 2024;8(11):igae085. 39790554 10.1093/geroni/igae085 PMC11714157

[130] Wijker C, Leontjevas R, Spek A, Enders-Slegers MJ. Process evaluation of animal-assisted therapy: feasibility and relevance of a dog-assisted therapy program in adults with autism spectrum disorder. Animals. 2019. 10.3390/ani9121103. 31835401 10.3390/ani9121103 PMC6940976

[131] Baumgartner E, Cho J-i. Animal-assisted activities for students with disabilities: obtaining stakeholders’ approval and planning strategies for teachers. Child Educ. 2014;90:281–90.

[132] Silcox D, Castillo YA, Reed BJ. The human animal bond: applications for rehabilitation professionals. J Appl Rehabil Counsel. 2014;45(3):27–35.

[133] Wintermantel L, Grove C. An evaluation of a dog-assisted social and emotional learning intervention in a school setting: study protocol for a cluster-randomised trial. Mental Health & Prevention. 2022;28:200246.

[134] McCullough A, Ruehrdanz A, Jenkins MA, Gilmer MJ, Olson J, Pawar A, et al. Measuring the effects of an animal-assisted intervention for Pediatric Oncology patients and their parents: a multisite randomized controlled trial. J Pediatr Oncol Nurs. 2017;35(3):159–77. 29268667 10.1177/1043454217748586

[135] De Santis M, Filugelli L, Mair A, Normando S, Mutinelli F, Contalbrigo L. How to measure human-dog interaction in dog assisted interventions? A scoping review. Animals. 2024;14(3):410. 38338052 10.3390/ani14030410 PMC10854530

